# Diversity of voltage-gated potassium channels and cyclic nucleotide-binding domain-containing channels in eukaryotes

**DOI:** 10.1038/s41598-020-74971-4

**Published:** 2020-10-20

**Authors:** Ilya Pozdnyakov, Pavel Safonov, Sergei Skarlato

**Affiliations:** Institute of Cytology, Russian Academy of Sciences, Saint Petersburg, 194064 Russia

**Keywords:** Phylogeny, Ion transport

## Abstract

Voltage-gated potassium channels (K_v_) and cyclic nucleotide-binding domain-containing cation channels HCN, CNG, and KCNH are the evolutionarily related families of ion channels in animals. Their homologues were found in several lineages of eukaryotes and prokaryotes; however, the actual phylogenetic and structural diversity of these ion channels remains unclear. In this work, we present a taxonomically broad investigation of evolutionary relationships and structural diversity of K_v_, HCN, CNG, and KCNH and their homologues in eukaryotes focusing on channels from different protistan groups. We demonstrate that both groups of channels consist of a more significant number of lineages than it was shown before, and these lineages can be grouped in two clusters termed K_v_-like channels and CNBD-channels. Moreover, we, for the first time, report the unusual two-repeat tandem K_v_-like channels and CNBD-channels in several eukaryotic groups, i.e. dinoflagellates, oomycetes, and chlorarachniophytes. Our findings reveal still underappreciated phylogenetic and structural diversity of eukaryotic ion channel lineages.

## Introduction

**Figure 1:**
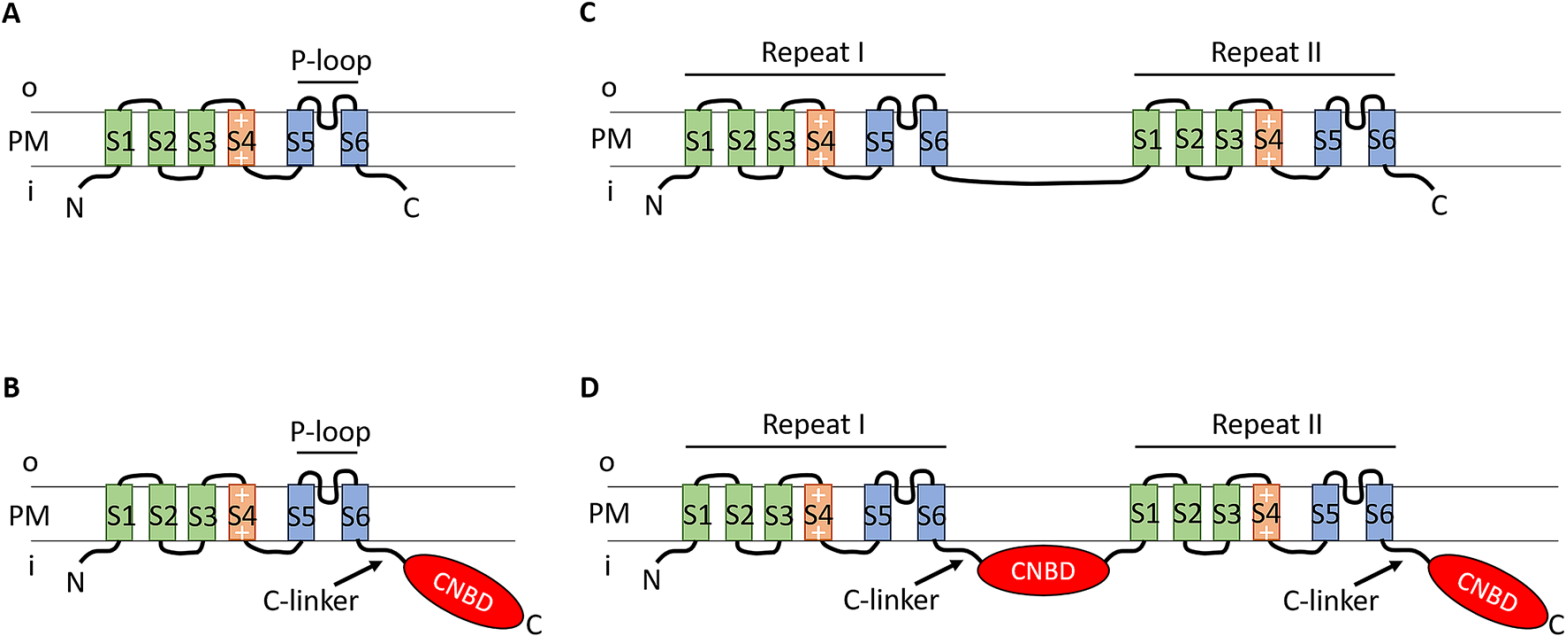
Schematic representation of the pore-forming subunits. (**A**) Voltage-gated potassium channel. (**B**) Cyclic nucleotide-gated channel. (**C**) Tandem voltage-gated potassium channel. (**D**) Tandem cyclic nucleotide-gated channel. Transmembrane segments S1–S4 form voltage-sensitive domains, whereas segments S5, S6, and P-loop between them contribute to a pore formation. *CNBD* cyclic nucleotide-binding domain, *i* intracellular compartment, *o* outer space, *PM* plasma membrane, *S1–S6* transmembrane segments. Segment S4 is rich in positive (+) amino acid residues.

Voltage-gated potassium channels (K_v_) and their close homologues, cyclic nucleotide-binding domain-containing channels, i.e., potassium channels KCNH, cyclic nucleotide-gated cation channels CNG, and hyperpolarization-activated cyclic nucleotide-gated cation channels HCN, belong to a large superfamily of transmembrane proteins called voltage-gated cation channel (VGCC) superfamily^1^. In addition to the ion channel families listed above, there are several more families within VGCC superfamily, including two families of the calcium-activated potassium channels, i.e., BK and IK/SK, which are closely related to the channels in focus^1^ but are not considered in this work. The core arrangement of a pore subunit of K_v_, KCNH, CNG, and HCN channels includes six transmembrane segments S1–S6 (6TM arrangement). Transmembrane segments S1–S4 form a voltage-sensitive domain, in which segment S4 functions as a voltage-sensor. A structural motif S5–P-loop–S6 forms a pore-domain (Fig. 1A,B). In a functional channel, four pore subunits are oligomerised, and four P-loops (one from each subunit) line a pore of a channel^2–5^. In addition, KCNH, CNG, and HCN channels possess a cyclic nucleotide-binding domain (CNBD) connected by C-linker to 6TM-structure at the C-terminus of each subunit (Fig. 1B). CNBDs of CNG and HCN channels can bind a molecule of cGMP or cAMP, respectively, and control gating of a channel^6^. CNBDs of KCNH channels bind cyclic nucleotides with low affinity and do not regulate channel opening^7^. Instead, the opening of these channels is regulated by an intrinsic ligand^8,9^. Besides, CNBD-containing channels—CNGK channels were identified in sperm cells of some invertebrates and fishes. The pore subunit of these potassium-selective channels represents a pseudo-tetrameric structure consisting of four 6TM–CNBD repeats^10,11^. In this research, KCNH, HCN, CNG, and CNGK channels, as well as their homologues, are referred to as CNBD-channels (CNBD-containing channels), whereas K_v_ channels of metazoans and their homologues in prokaryotes and other eukaryotes—as K_v_-like channels.

CNBD-channels and K_v_-like channels are involved in many essential physiological processes in different cell types of metazoans. The opening of K_v_ channels during membrane depolarization ensures the repolarization phase of the action potential in excitable cells^12^. HCN channels play a crucial role in the regulation of rhythmical activity of cardiomyocytes and neurons, and CNG channels are involved in the receptor potential formation during the light perception by retina cells^13^. Moreover, KCNH channels regulate the firing of neurons^14^, and CNGK channels are involved in sperm chemotaxis in fishes and some invertebrates^10,11^. In plants, CNGC channels are implicated in the calcium entry and pathogen defense response, and potassium-selective ‘K_v_-like’ channels participate in stomatal closure (GORK channel) and potassium transport in xylem (SKOR channel)^15^. Both plant CNGC and ‘K_v_-like’ channels are now should be considered as CNBD-channels^16^.

**Figure 2:**
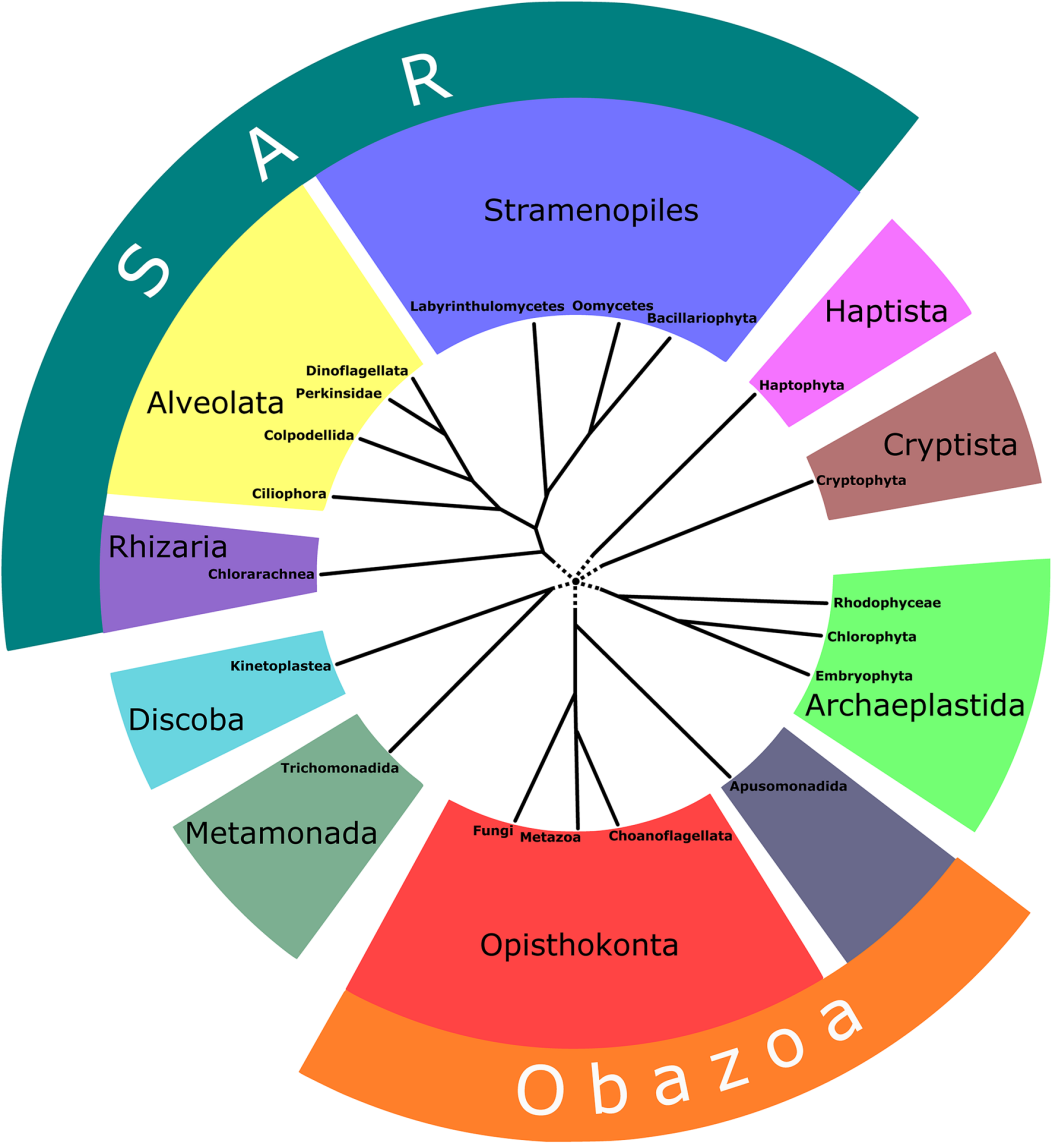
Schematic representation of modern views on the phylogenetic relationships between eukaryotic lineages considered in this study (based on Adl et al.^27^ and Keeling and Burki^28^).

In addition to metazoan and plant CNBD-channels, CNBD-channel genes were found in other eukaryotic groups: Ciliophora^17^, Chlorophyta^18^, and Bacillariophyta^18^, Choanoflagellata^19^, Dinoflagellata^20^, and Oomycetes^21^ (Fig. 2). Additionally, CNBD-channels were identified in bacteria and were characterised as potassium-selective cyclic nucleotide-gated channels^22,23^, but their physiological role in these organisms remains enigmatic.

K_v_-like channels are an ancient group of ion channels that were identified in genomes and transcriptomes of bacteria, archaea, and many eukaryotes^17,20,24,25^. However, true K_v_-like channels have not been identified in land plants^16^ and believed to be uncommon in fungi^26^. An ancient K_v_-like channel is considered to be a basal structure for CNBD-channels^25^ which probably represent a result of a fusion event between the K_v_-like channel gene and CNBD motif. It should be highlighted that the physiological role of both groups of ion channels in protists and prokaryotes has not been understood yet.

In the present work, we investigated the phylogeny of K_v_-like channels and CNBD-channels of eukaryotes, including diverse protistan groups (Fig. 2), and their structural diversity with an emphasis on the arrangement of the pore-forming subunits, which should improve our understanding of the evolution of these vital ion channels.

## Results

### Overall structural diversity and phylogeny of K_v_-like channels and CNBD-channels

During the database searches, 262 homologues of K_v_, KCNH, CNG, and HCN channels from 19 groups of eukaryotes, as well as several bacterial and archeal channels were chosen for the phylogenetic analysis (269 homologues and 302 sequences including separated repeats of the tandem channels) (Supplementary Table S1).

The analysis of the primary structure of these sequences predicted that most of the channels considered in this work possess conventional 6TM (for K_v_-like channels) and 6TM-CNBD (for CNBD-channels) arrangement of the pore subunit. In the previous work, we identified the unusual long transcripts of K_v_ and HCN/CNG-homologues in two transcriptomes of the dinoflagellate *Prorocentrum minimum*. The subsequent analysis revealed the presence of the tandem repeat 6TM and 6TM-CNBD structures in the predicted proteins^20^. Here, we report the presence of both two-repeat tandem HCN/CNG-like and K_v_-like sequences in transcriptomes of other dinoflagellate species, as well as in genomes and transcriptomes of different oomycete species. Moreover, tandem K_v_-like channels were found in the transcriptome of the chlorarachniophyte *Lotharella globosa* (Fig. 1C,D). For further phylogenetic analyses, all these tandem channels were cut in two 6TM-repeats that were added to the alignment as separate sequences.

**Figure 3:**
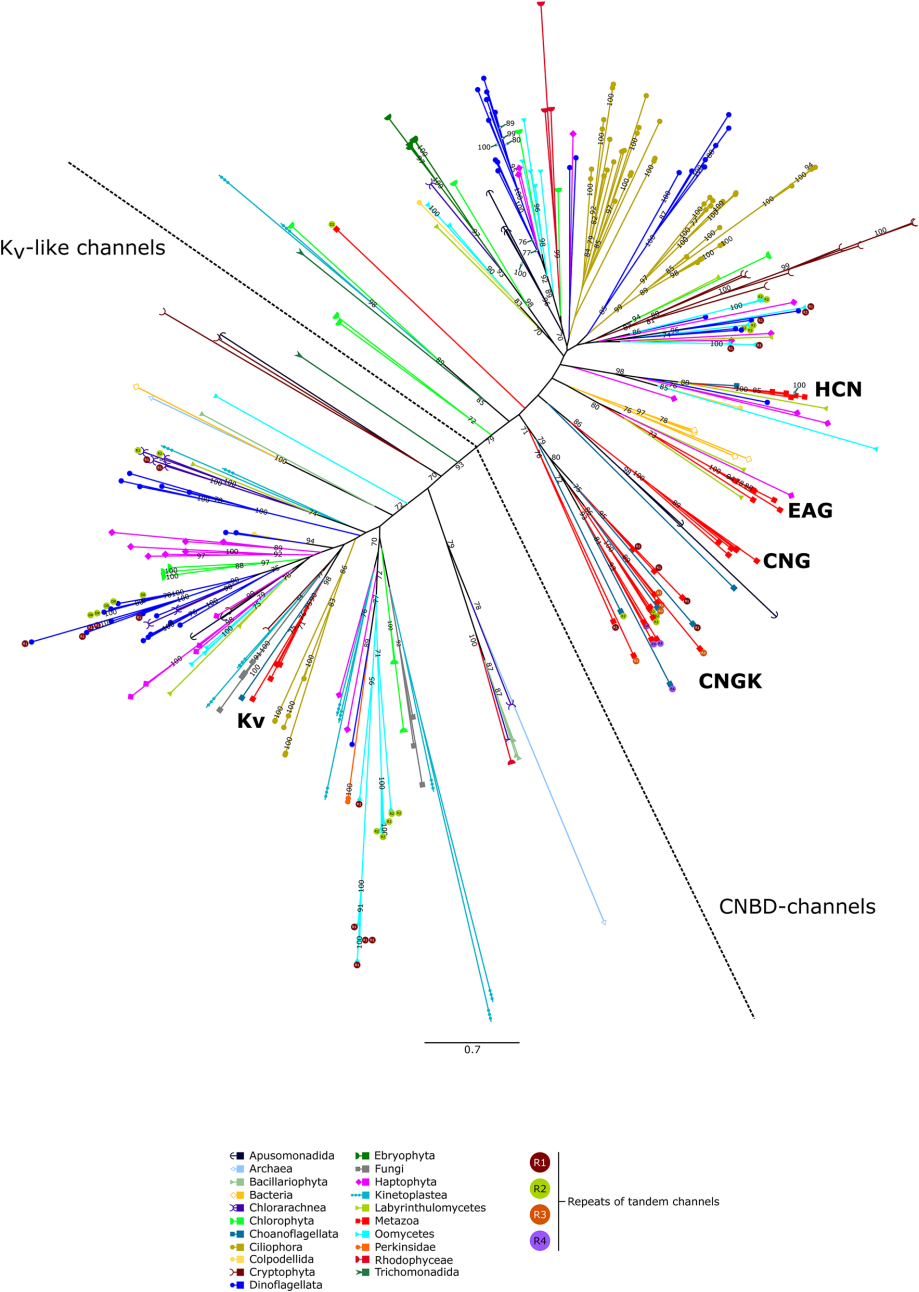
Unrooted phylogenetic tree of K_v_-like channels and CNBD-channels inferred using maximal likelihood analysis (LG + F + R8 model, 10,000 ultrafast bootstrap replicates). The numbers on branches show bootstrap values (not shown when supports < 70). The dotted line delimits K_v_-like and CNBD-channels. The alignment is provided in Supplementary Dataset S1.

The overall phylogeny of K_v_-like and CNBD-channels inferred by means of the maximal likelihood analysis showed a clear tendency of the separation of these two groups of channels on the tree (Fig. 3). However, it should be underlined that in the resulting alignment, the number of sequences (302) was much greater than the number of positions (196). Consequently, the tree shows only the general trend of the distribution of these two large groups of channels, but not the exact branching order of the individual clades, despite high bootstrap values in some cases. Therefore, we further performed the phylogenetic analysis of K_v_-like and CNBD-channels separately, thus reducing the number of sequences and increasing the number of conservative positions in the alignments.

### Phylogenetic diversity of CNBD-channels

**Figure 4:**
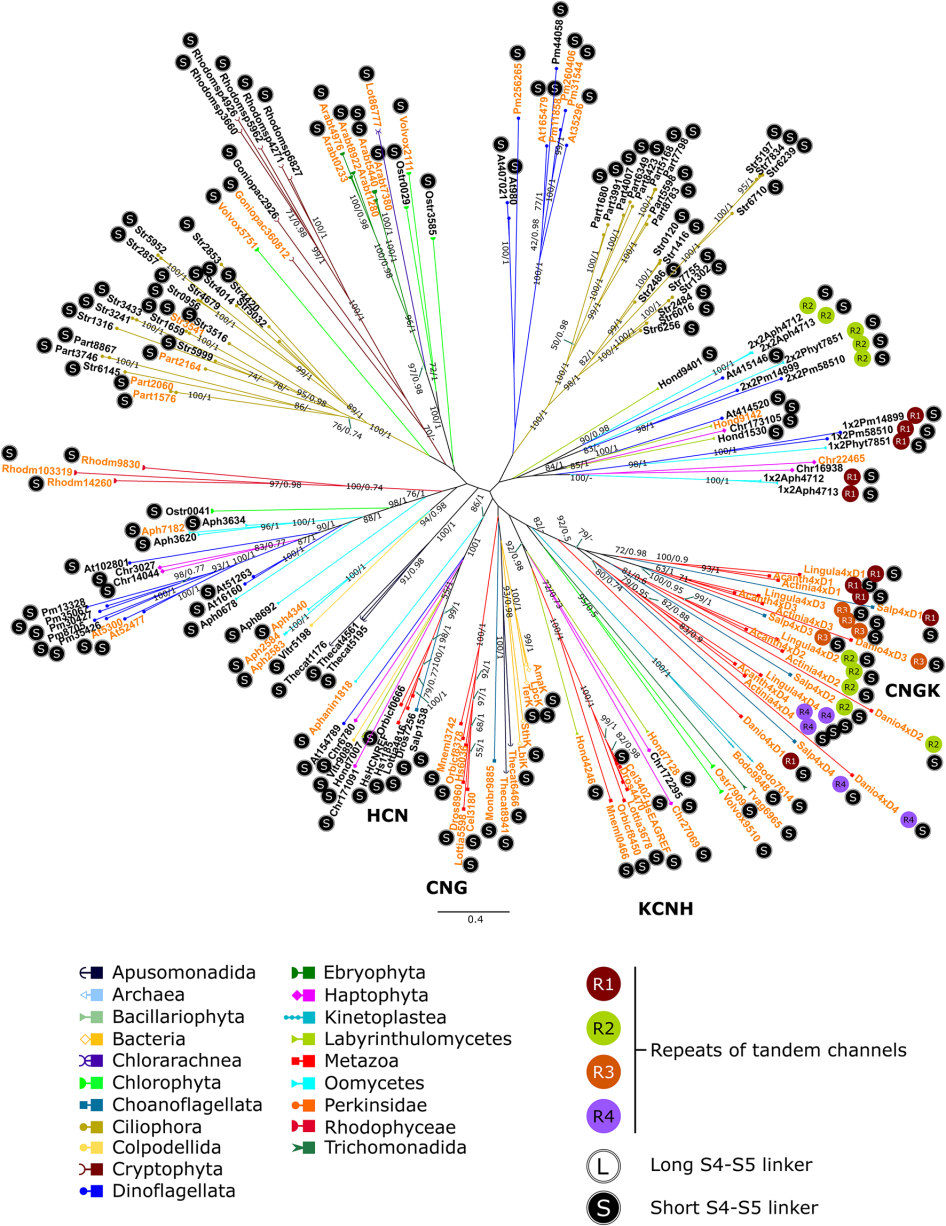
Unrooted phylogenetic tree of CNDB-channels inferred using maximal likelihood analysis (LG + F + R8 model, 10,000 ultrafast bootstrap replicates). The numbers on branches show bootstrap values and Bayesian posterior probabilities, respectively (not shown when supports < 70/0.90). Sequences with lysine/arginine-poor S4 are highlighted in orange. For sequence abbreviations see Supplementary Table S1. The alignment is provided in Supplementary Dataset S2.

In order to infer phylogenetic trees of CNBD-channels more accurately, we carried out the maximal likelihood and Bayesian analyses. Both methods of the phylogeny reconstruction were used for each of the three datasets: (1) without an outgroup (Fig. 4), (2) containing the sequence KvAP (K_v_-like channel of the archaeon *Aeropyrum pernix*) as an outgroup (Supplementary Fig. S1), and (3) containing the sequence HsKvREF (K_v_1.7 channel of *Homo sapiens*) as an outgroup (Supplementary Fig. S2).

Remarkably, the canonical animal families of CNBD-channels, i.e., HCN, CNG, and KCNH, do not form a single clade of metazoan CNBD-channels. Instead, there are three independent clades in every variant of the phylogenetic analysis. However, HCN channels of metazoans cluster with CNBD-channels of diaphoretickes: a colpodellid, a dinoflagellate, a labyrinthulomycete, and a haptophyte, with strong supports 100/1 (except the tree with HsKvREF as an outgroup where respective supports were 100/0.84).

Four-repeat tandem CNGK channels of metazoans and choanoflagellates form a distinct topological clade in every variant of the phylogenetic analysis. The first repeats are closely related to the third repeats, and the second repeats are closely related to the fourth repeats. The only exception is the distant location of the first repeat of the *Danio rerio* CNGK channel, which is most likely an artifact related to the absence of some conservative positions present in other CNGK sequences (Supplementary Dataset S2).

The first and second repeats of the two-repeat tandem CNBD-channels of oomycetes and dinoflagellates form four clades (Fig. 4): (1) clade containing the first repeats of the oomycete *Aphanomyces invadans* channels, (2) clade containing the second repeats of *A. invadans* channels, (3) clade containing the first repeats of the dinoflagellate *Prorocentrum minimum* channels*,* and (4) clade containing the second repeats of the dinoflagellate *P. minimum* channels. The sequences (both the first and second repeats) of the oomycete *Phytophthora infestans* cluster with the sequences of *P. minimum* without strong supports (Fig. 4, Supplementary Figs. S1, S2). Therefore, the position of the *Ph. infestans* tandem channel repeats on the trees may represent an artifact. The distribution of the parts of the tandem sequences is not random: the first and second repeats cluster in their own clades. Branching in the clades of the first repeats is similar to that in the clades of the second ones. In addition, the phylogeny reconstruction was performed for the tandem CNBD-channel sequences containing both repeats, which allowed us to include more conservative positions in the analysis (Supplementary Fig. S3). The resulting phylogeny demonstrated a similar branching.

### Phylogenetic diversity of K_v_-like channels

**Figure 5:**
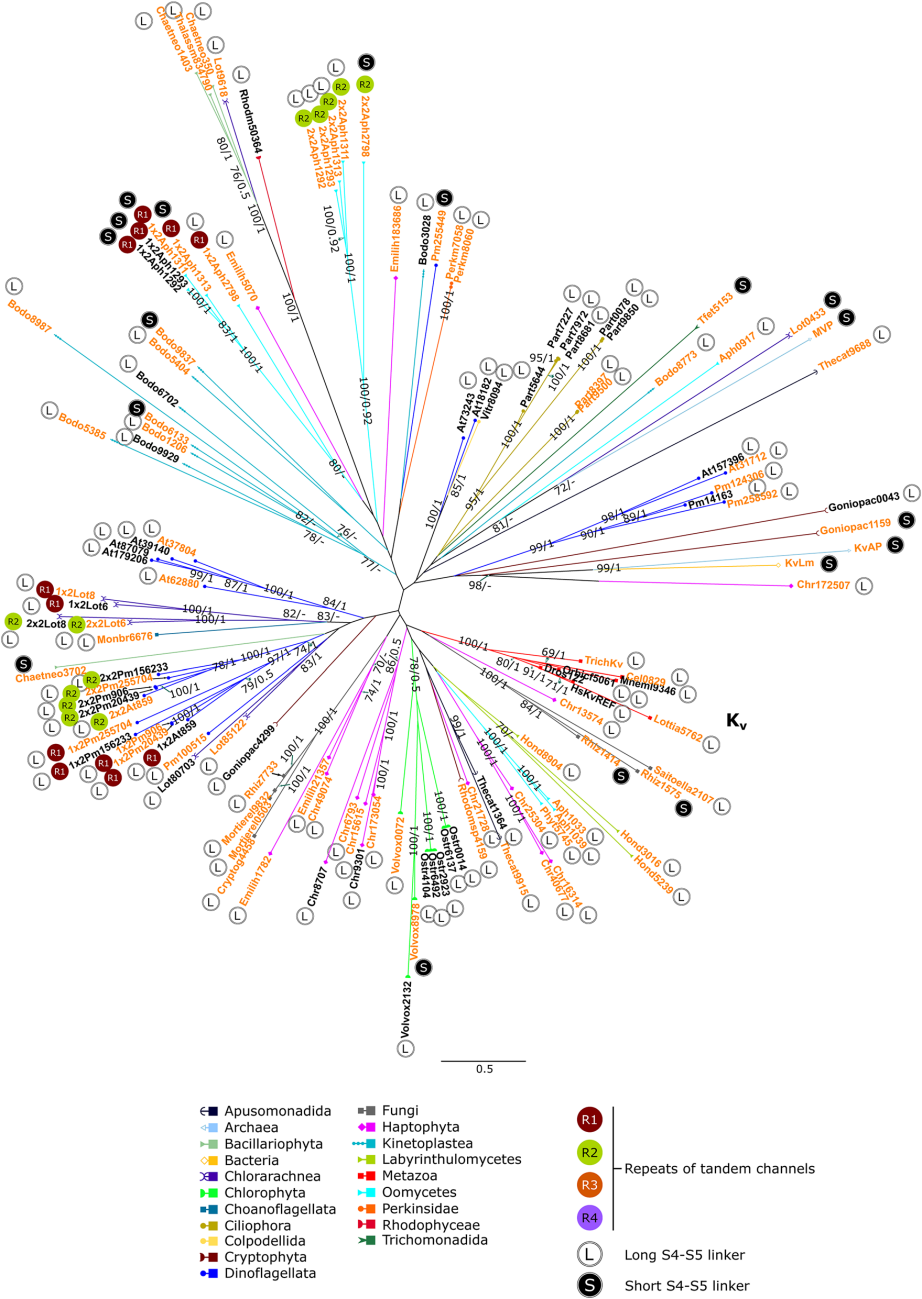
Unrooted phylogenetic tree of K_v_-like channels inferred using maximal likelihood analysis (LG + F + R5 model, 10,000 ultrafast bootstrap replicates). The numbers on branches show bootstrap values and Bayesian posterior probabilities, respectively (not shown when supports < 70/0.90). Sequences with lysine/arginine-poor S4 are highlighted in orange. For sequence abbreviations see Supplementary Table S1. The alignment is provided in Supplementary Dataset S6.

We performed three variants of both maximal likelihood and Bayesian analyses to reconstruct the phylogeny of K_v_-like channels: (1) without an outgroup (Fig. 5), (2) with LbiK (CNBD-channel of the bacterium *Leptospira biflexa*) as an outgroup (Supplementary Fig. S4), and (3) with HsHCNREF (HCN1 channel of *Homo sapiens*) as an outgroup (Supplementary Fig. S5). The results of the analyses demonstrate that K_v_ channels of metazoans represent one of many separate lineages of K_v_-like channels; other eukaryotes possess their own K_v_-like channels which are not closely related to those of metazoans. Surprisingly, K_v_-like channels of fungi (here we used representatives of Basidiomycetes, Ascomycetes, Glomeromycetes, and Mucoromycetes) form two distinct clades indicating that they possess at least two subfamilies of K_v_-like channels.

Similar to the tandem CNBD-channels, the tandem K_v_-like channels are not grouped randomly: the first and second repeats form their own clades. Moreover, clades of the first and second repeats form at least topological clusters in accordance with the organism phylogeny. There are distinct clusters of the oomycete, dinoflagellate, and chlorarachniophyte tandem channels (Supplementary Figs. S4, S5). The dinoflagellate tandem channels are topologically close to the chlorarachniophyte tandem channels, whereas the oomycete tandem channels are positioned separately. This finding was supported by the phylogeny reconstruction implementing sequences containing both repeats in order to increase the number of conservative sites in the alignment (Supplementary Fig. S6).

### Characterization of channel functional determinants

#### Selectivity filter

Both CNBD-channels and K_v_-like channels possess the conservative consensus selectivity filter motif TVGY/FG. Other polar amino acid residues, serine (S) or cysteine (C), replace threonine (T) in some cases. Non-polar amino acid residues often replace valine (V) (Supplementary Table S2). Significant deviations from the consensus sequence are rare and represent a feature of individual channels rather than clades. The clade of metazoan CNG channels with the TIGE selectivity filter represents the only one exception.

#### Voltage sensor S4

**Figure 6:**
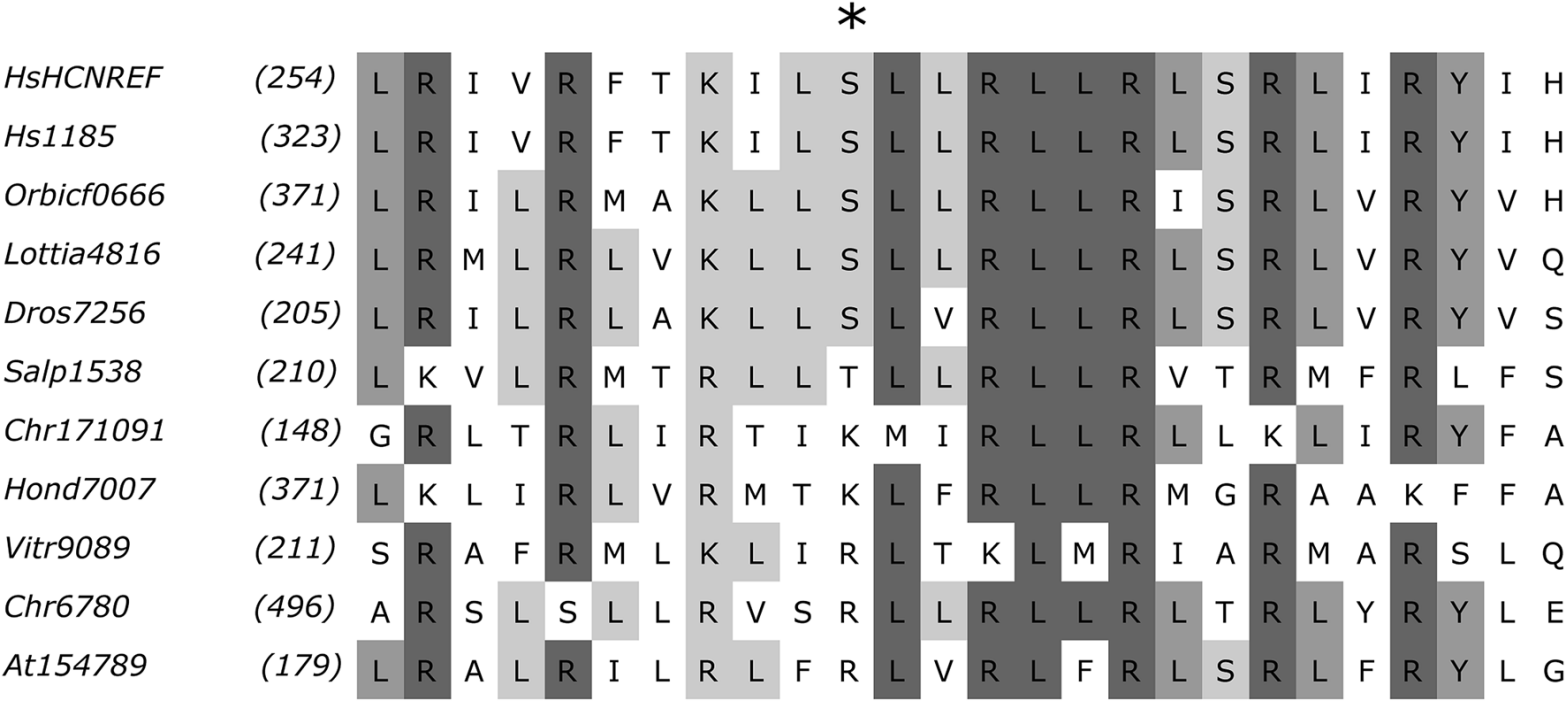
Multiple alignment of transmembrane segment S4 of HCN channels and protistan CNBD-channels clustering with them on the phylogenetic trees. Asterisk indicates the middle position of S4 which can be occupied by polar amino acid residues (S/T) or positively charged amino acid residues (R/K). At154789—CNBD-channels of *Alexandrium tamarense* (Dinoflagellata). Chr171091, Chr6780—CNBD-channels of *Chrysochromulina polylepis* (Haptophyta). Dor7256—HCN channel of *Drosophila busckii* (Metazoa). HsHCNREF, Hs1185—HCN channels of *Homo sapiens* (Metazoa). Hond7007—CNBD-channel of *Hondaea fermentalgiana* (Labyrinthulomycetes). Lottia4816—HCN channel of *Lottia gigantea* (Metazoa). Orbicf0666—HCN channel of *Orbicella faveolata* (Metazoa). Salp1538—HCN channel of *Salpingoeca rosetta* (Choanoflagellata). Vitr9089—*Vitrella brassicaformis* (Colpodellida).

The voltage sensors containing seven positively charged amino acid residues of arginine (R) and lysine (K) with a polar residue of serine (S) or threonine (T) replacing one of R/K in the middle of a segment are often considered as a feature of HCN channels^6,19^. The analysis of the primary structure of K_v_-like channels and CNBD-channels shows that long segments S4 bearing seven and more R/K residues spread in both channel groups (Supplementary Table S2). Clades with R/K-rich S4s are more common among protistan CNBD-channels than in K_v_-like channels. The mapping of R/K-richness on the phylogenies shows that there are R/K-rich, R/K-poor, and mixed channel clades in both channel groups (Figs. 4, 5). At the same time, our results show that the described feature is characteristic of the metazoan and choanoflagellate HCN channels only. The voltage sensors of the protistan channels grouped with HCN channels have 7–8 positive charges but do not have a replacement of R/K by S/T in the middle of a segment (Fig. 6). Replacement of R/K in the homologous position by polar or even non-polar amino acid residues occurred in some other cases but without a link to the phylogenetic position of a sequence.

Remarkably, in many cases of two-repeat tandem K_v_-like channels, but not in the case of tandem CNBD-channels, one of the repeats bear more positive charges than the other (Fig. 5). For instance, the first repeat of Aphanomyces tandem K_v_-like channel (1 × 2Aph1292) has eight positive charges against five in the second repeat (2 × 2Aph1292).

#### S4–S5 linker

An intracellular protein loop between S4 and S5 links a voltage-sensitive domain with a pore-domain of a channel and conducts conformational changes from a voltage sensor to a pore^29^. According to the structural data, the length of S4–S5 linker affects the architecture of the whole channel complex, and two possible channel arrangements exist, the swapped (with a long linker) and non-swapped (with a short linker)^6^. Remarkably, all CNBD-channels considered in this work contain relatively short (less than ten amino acid residues, 4.4 ± 1.5 in our data set) S4–S5 linkers (Fig. 4). In contrast, K_v_-like channels are generally characterised by a longer (11.1 ± 2.2 in our data set) sequence of a linker. However, some channels underwent the secondary shortening of this structure, which happened several times independently (Fig. 5, Supplementary Table S2). All of the oomycete two-repeat tandem K_v_-like channels considered in this work possess both short (in the first repeat) and long (in the second repeat) linkers in the sequences. At the same time, other tandem channels bear only one type (short or long) of linkers in each repeat.

#### Cyclic nucleotide-binding domain

**Figure 7:**
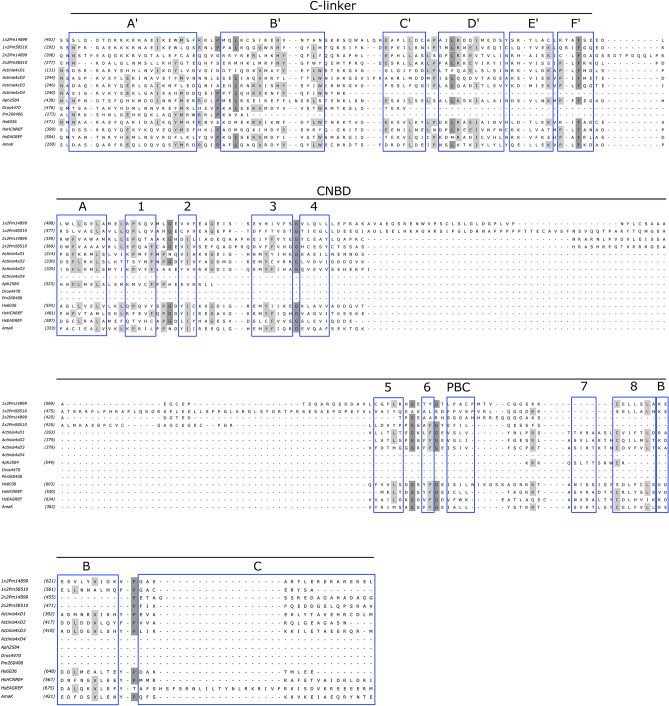
Multiple alignment of C-linker and cyclic nucleotide-gated domain (CNBD) of CNBD-channels. Regions corresponding to the secondary structures of C-linkers and CNBDs of metazoan and bacterial CNBD-channels are boxed. A′–F′, A–B—α-helixes, PBC—phosphate-binding cassette, 1–8—β-strands. 1 × 2Pm14899, 1 × 2Pm58510—the first repeats of tandem CNBD-channels of *Prorocentrum minimum* (Dinoflagellata). 2 × 2Pm14899, 2 × 2Pm58510—the second repeats of respective tandem channels. Actinia4xD1, Actinia4xD2, Actinia4xD3, Actinia4xD4—four repeats of CNGK channel of *Actinia tenebrosa* (Metazoa). Aph2584—CNBD-channel of *Aphanomyces invadans* (Oomycetes). Dros4470—KCNH channel of *Drosophila melanogaster* (Metazoa). Pm260406—CNBD-channels of *P. minimum* (Dinoflagellata). Hs6036, HsEAGREF, HsHCNREF—CNG, KCNH, and HCN channels of *Homo sapiens* (Metazoa), respectively. AmaK—CNBD-channel of *Arthrospira maxima* (Bacteria).

Cyclic nucleotide-binding domain (CNBD) and C-linker connecting CNBD with the pore domain of a channel are present only in CNBD-channels (Supplementary Table S2). However, in some cases, partial or complete deletion of C-linker and CNBD took place. The sequences Pm260406 (dinoflagellate *Prorocentrum minimum*), Actinia4xD4 (the fourth repeat of CNGK channel of the cnidarian *Actinia tenebrosa*), and Dros4470 (KCNH channel of the fruit fly *Drosophila melanogaster*) lack a substantial part of C-region including CNBD and/or C-linker, most likely due to sequence incompleteness. Nevertheless, a real deletion could take place. Some sequences lack internal parts of C-region. Single-repeat CNBD-channel of the oomycete *Aphanomyces invadans* Aph2584 has a substantial deletion in C-linker and most parts of CNBD, including those participating in the formation of a cyclic nucleotide-binding site (β-strands 5–8, a phosphate-binding cassette (PBC), and α-helix C^30^), i.e. the helixes C’–F’, β-strands 5–8, PBC, and a helix B. Tandem channels of the dinoflagellate *Prorocentrum minimum* also have CNBD with deletions of some structures which take part in the cyclic nucleotide binding. Two-repeat tandem channel 2Pm14899 lacks β-strand 7 in the first repeat CNBD and β-strands 5, 7, and 8 along with helix B in the second repeat CNBD. Two-repeat tandem channel 2Pm58510 lacks β-strand 7 in the first repeat CNBD and β-strands 7,8, and a helix B in the second repeat (Fig. 7). Albeit sequence incompleteness arising from sequencing and assembly errors cannot be excluded, a similar pattern of reduction in different two-repeat tandem channel sequences of *P. minimum* from the same channel clade is indicative of the real deletions.

## Discussion

The phylogenetic analysis revealed that all ion channels considered in this work can be assigned to one of the two groups: (1) K_v_-like channels containing metazoan K_v_ channel family among other K_v_-like channel clades, and (2) CNBD-channels containing familiar HCN, CNG, KCNH, and CNGK channels among other CNBD-channel clades (Fig. 3). The K_v_-like and CNBD channel branches are not mixing with each other on the obtained tree. According to the bootstrap values, K_v_-like channels are likely a monophyletic group of ion channels. At the same time, the uncertainty in CNBD-channel branching does not allow us to make a definite conclusion regarding the monophyletic or polyphyletic origin of this channel group. In other words, based on the tree we cannot judge whether eukaryotic CNBD-channels have a single origin or they emerged in various lineages independently as a result of de novo evolution and/or horizontal gene transfer (HGT).

Both CNBD-channels and K_v_-like channels show a great phylogenetic diversity: classical K_v_, HCN, CNG, and KCNH channels, as well as four-repeat CNGK channels of animals, represent five families among a variety of other families of channels belonging to different eukaryotic organisms (Fig. 3). Earlier we demonstrated that the eukaryotic four-domain voltage-gated cation channels (FVCC) are also very diverse^31^. These data point at a still underappreciated diversity of the eukaryotic ion channel lineages.

Remarkably, the metazoan CNBD-channels do not form a single metazoan clade. Instead, all four channel families seem to be evolutionary independent (Fig. 4). This observation may be rooted in both the complex evolutionary history (e.g., horizontal gene transfer, high rate of evolution) of these proteins and the scarcity of available data. Recently, Jegla et al.^16^ analysed the phylogeny of the plant voltage-gated potassium channels which turned out to be a part of CNBD-channels and proposed that the eukaryotic CNBD-channels are a result of an HGT from bacteria to eukaryotes. The HGT hypothesis is supported by the fact that there is still no evidence of CNBD-channels in archaea. In this study, we identified the clade consisting of the metazoan HCN and CNBD-channels of eukaryotes very distantly related to animals (Fig. 4, Supplementary Figs. S1, S2). It seems unlikely that in the course of evolution, such non-conservative proteins as ion channels could undergo so little changes that they form clades unifying distantly related groups, i.e., animals and alveolates or haptophytes. Therefore, if this grouping reflects the true evolutionary relationship between HCN and protistan CNBD-channels, then it could represent a case of HGT between these eukaryotic lineages. Nevertheless, it should be taken into account that the future sequencing of protistan genomes and transcriptomes will result in the identification of more ion channel sequences in various eukaryotic lineages. The addition of those presumable, yet undiscovered sequences to the alignments theoretically can affect the phylogenetic clustering and respective supports of different nodes.

A distinct position of four-repeat CNGK channels of metazoans and choanoflagellates gives rise to the question about a single-repeat ancestor of these channels. None of CNG, HCN, or KCNH channels can be proposed for this role based on our data. First, there are no supported nodes uniting these clades of ion channels. Second, these clades do not have close topological positions in all variants of phylogenetic reconstruction (Fig. 4, Supplementary Figs. S1, S2). The branching order of separated repeats suggests that the first repeats are more homologous to the third ones, and the second repeats are more homologous to the fourth ones. This implies two sequential rounds of the ancestor gene duplication similarly to the evolutionary history of FVCCs^32^.

The phylogenetic diversity of the fungal K_v_-like channels (Fig. 5) is of particular interest because fungi, unlike metazoans, have lost many genes of ion channels^33^ and are believed to be poor in the K_v_-like channel homologues^26^. Here we demonstrate that fungi possess two phylogenetically distinct subfamilies of K_v_-like channels. Remarkably, Liebeskind et al.^33^ reported that *Rhizophagus* (Glomeromycetes) and *Mortierella* (Mucoromycetes), which were considered in the present work too, nevertheless had a little net gain in ion channel genes. We suppose that this net gain can be partially due to gains in K_v_-like channel content.

Both K_v_-like channels and CNBD-channels show a high degree of conservation of the selectivity filter motif. In most channel lineages, the motif sequence is close to consensus TVGY/FG sequence that should reflect a pore structure of the ancestor channels. The non-canonical selectivity filters of such channel clades as metazoan CNG and plant CNGC (the plant non-selective cationic CNBD-channels^16^ which were not included in the analysis) likely evolved from TVGY/FG-motif.

Previously, Cai^19^ demonstrated that a specific feature of HCN family, the long R/K-rich S4s in which R/K in the middle is replaced by polar serine or threonine, evolved before the divergence of animals and choanoflagellates. Since we did not find CNBD-channels with such S4s in Holomycota, the lineage sister to Holozoa, and in Apusomonadida, our results confirm that modified S4s and HCN family itself likely emerged in Holozoa. Moreover, if the grouping of holozoan HCN channels with the CNBD-channels of a colpodellid, a dinoflagellate, a labyrinthulomycete, and a haptophyte reflects true evolutionary relationships, the voltage sensors of HCN channels could have evolved from the long S4s without a replacement of R/K by S/T.

The eukaryotic CNBD-channels are characterised by a short S4–S5 linker and the presence of a C-linker–CNBD structure (Fig. 4, Supplementary Table S2). Since S4–S5 linkers of the metazoan CNBD-channels likely interact with C-linkers participating in channel gating^34,35^, the shortening of this segment in the course of evolution could be a result of the protein adaptation to the appearance of a new functional domain, i.e. CNBD. Furthermore, it was shown that the voltage-sensitive-domains of KCNH channels are able to mediate gating with a broken peptide bond in S4–S5 linkers^36,37^. Thus, the shortening of the linker may reflect a change in functional significance of this protein region for the gaiting process in the course of evolution. In addition, available structural data show that ion channels with short S4–S5 linkers have non-swapped arrangement^6^. Hence, we suggest such an arrangement as the ancestral state for the entire CNBD-channel group. The fact that known bacterial CNBD-channels except MloK1 show non-swapped arrangement supports this suggestion^38^. In contrast, K_v_-like channels usually possess longer S4–S5 linkers (Fig. 5, Supplementary Table S2). However tandem K_v_-like channels of oomycetes attract attention since they possess both short and long linkers in the same sequence. This implies an unusual asymmetric architecture of these channels with mixed swapped and non-swapped arrangement of the pore subunits, but decisive conclusions can only be made based on the experimentally obtained three-dimensional structures.

In the present work, we for the first time, report the presence of the two-repeat tandem CNBD-channels and K_v_-like channels in diverse eukaryotic species. In all cases, the first and second repeats of the tandem channels form separate clades but not the mixed ones. Nonrandom distribution of both sequence parts on the phylogenetic trees indicates that they are not the products of polycistronic pre-mRNAs, as it theoretically could be in the case of dinoflagellates^39^. The low degree of identity (30% and less) between the first and second repeats, the difference in the R/K content of the repeats of tandem Kv-like channels, the difference of S4–S5 linker length of the repeats in some of tandem CNBD-channels, the partial reduction of CNBD in one of the repeats of some tandem CNBD-channels, as well as the fact that branching of the first repeats corresponds to the branching of the second ones (Fig. 4) suggest the tight co-evolution of both parts of a tandem protein accompanied by specialization of each repeat.

The two-repeat tandem CNBD-channels of dinoflagellates and oomycetes form two phylogenetic clusters (Supplementary Fig. S3). Similarly, the tandem K_v_-like channels of dinoflagellates, oomycetes, and chlorarachniophytes form three phylogenetic clusters (Supplementary Fig. S6). All these groups of organisms are evolutionary related and represent lineages of the supergroup SAR^27,28^ (Fig. 2). However, we did not identify tandem channels in other SAR groups, including those closely related to dinoflagellates (apicomplexans and ciliates), oomycetes (e.g. labyrinthulomycetes, phaeophytes, and diatoms), and chlorarachniophytes (e.g., foraminifera). Therefore, it is possible that these channels represent five independent cases of gene duplication or fusion. Nevertheless, it should be noted that the tandem CNBD-channels of dinoflagellates and oomycetes are located in the same parts of the trees (Fig. 4, Supplementary Figs. S1, S2), and they have similar structural features such as R/K-richness of S4s and length of S4–S5 linkers. Likewise, tandem K_v_-like channels of dinoflagellates and chlorarachniophytes hold close position in the trees (Fig. 5, Supplementary Figs. S4, S5), and share structural features such as long S4–S5 linkers and variability of R/K-content in S4s. Thus, it is also possible that our phylogenetic analysis has not enough strength to resolve the relationship between these channels.

The selectivity filter sequences of the two-repeat tandem channels are represented by the TVGYG motif or close variants. This allows us to suggest that such channels are at least potassium-permeable. It is known that potassium-selective channels, such as K_v_, and non-selective cation channels, such as HCN, possess close variants of the selectivity filter motif (TVGYG for K_v_; CIGYG for HCN). At the same time, structural data show that three-dimensional structures of the selectivity filters of K_v_ and HCN channels are different^2,4^. Thus, the selectivity of these channels cannot be predicted from the primary protein structure since it depends on the coordination bonds formed by the carbonyl oxygens in a three-dimensional structure. Partial reductions of CNBDs of the first or second repeat of dinoflagellate tandem CNBD-channels (Fig. 7) indicate that they cannot bind cyclic nucleotides and probably function as voltage-gated cation channels. Since two-repeat tandem channels of oomycetes possess full-length C-linkers and CNBDs, cyclic nucleotide-gating of these channels can be expected.

Tandem channels have been previously described in VGCC superfamily^25^. Potassium leak channels (K_v2P_) represent a dimer of tandem modules consisting of S1–P-loop–S2 homologous to S5–P-loop–S6 motif of K_v_-like channels and CNBD-channels. Potassium channels TOK found in fungi have the α-subunits formed by the 6TM and S1–P-loop–S2-like motifs in tandem. TOK channels are considered to be a result of a fusion between K_v_-like channel and inwardly rectifying potassium channel (K_ir_) genes^25^. The pore subunit of a so-called two-pore calcium channel (TPC) also represents a tandem of two 6TM motifs. FVCC α-subunit (e.g., voltage-gated calcium and sodium channels) consists of four homologous 6TM repeats. All these channel families are believed to be products of gene duplication events. Our results corroborate the idea that internal oligomerization (formation of tandem structures) of a channel was more common among VGCCs than previously thought and occurred many times independently.

The physiological role of the new type of tandem channels described here is a subject of future research. Tandem channels of parasitic oomycetes are of particular interest since these unusual channels are not present in their hosts, both animals and plants. Therefore, they can be considered as potential targets for chemicals inhibiting the growth of oomycetes.

## Materials and methods

### Database search for homologues of K_v_-like channels and CNBD-channels

Homologues of human K_v_, HCN, CNG, and KCNH channels were found using blastp search in the following databases: Marine Microbial Eukaryote Transcriptome Sequencing Project^40^ (MMETSP; https://data.imicrobe.us/project/view/104, Combined Assemblies), GenBank, and RefSeq. To identify more unusual tandem sequences homologous to K_v_ and CNBD-channels, we used the previously found tandem sequences of the dinoflagellate *Prorocentrum minimum*^20^ as queries. Prokaryotic sequences were taken from Santos et al.^41^ and Brams et al.^23^. The full list of channel homologues contained 302 sequences, including separated repeats of the tandem channels (Supplementary Table S1).

### Multiple sequence alignment

The dataset containing amino acid sequences of K_v_-like and/or CNBD-channels, as well as their homologues was aligned by means of MAFFT7^42^. The results of the multiple alignments were improved manually and used in the primary structure analysis. The multiple alignments were visualised in Unipro UGENE^43^.

### Phylogeny reconstruction

Non-conservative sites were manually removed from the obtained alignments. In the cases when the alignment contained both K_v_-like channels and CNBD-channels, the non-conservative domains in N- and C-termini, including C-linkers and CNBDs, were removed entirely. The resulting alignments (Supplementary Datasets S1–S9) were used to select the evolutionary model utilizing ProtTest^44^ for Bayesian analysis and IQ-tree^45^ (which allows testing free rate models) for maximal likelihood analysis according to the Bayesian information criterion (Table 1). Maximal likelihood phylogenetic analysis was performed by means of IQ-tree^45^ with ultrafast bootstrapping (10,000 replicates). Bayesian analysis was conducted in MrBayes 3.2.6^46^ with 10 million generations, two runs, four Markov chains, and sampling of every 5000 chains. All phylogenetic reconstructions were made in CIPRES Science Gateway^47^. Phylogenetic trees were visualised in FigTree 1.4.2^48^.

**Table 1:** List of the evolutionary models used in the maximal likelihood (ML) and Bayesian (BA) phylogenetic tree calculations.

Phylogenetic tree	Evolutionary model for ML	Evolutionary model for BA
Kv-like channels and CNBD-channels, unrooted (Fig. 3)	LG + F + R8	–
CNBD-channels, unrooted (Fig. 4)	LG + F + R8	LG + F + Г_4_
CNBD-channels, KvAP is an outgroup (Supplementary Fig. S1)	LG + F + R7	LG + F + Г_4_
CNBD-channels, HsKvREF is an outgroup (Supplementary Fig. S2)	LG + F + R7	LG + F + Г_4_
Two-repeat tandem CNBD-channels (Supplementary Fig. S3)	LG + Г_4_	LG + Г_4_
Kv-like channels, unrooted (Fig. 5)	LG + F + R5	LG + F + I + Г_4_
Kv-like channels, LbiK is an outgroup (Supplementary Fig. S4)	LG + F + R5	LG + F + I + Г_4_
Kv-like channels, HsHCNREF is an outgroup (Supplementary Fig. S5)	LG + F + R5	LG + F + I + Г_4_
Two-repeat tandem Kv-channels (Supplementary Fig. S6)	LG + F + Г_4_	LG + F + Г_4_

## Supplementary information

Supplementary Dataset S1.

Supplementary Dataset S2.

Supplementary Dataset S3.

Supplementary Dataset S4.

Supplementary Dataset S5.

Supplementary Dataset S6.

Supplementary Dataset S7.

Supplementary Dataset S8.

Supplementary Dataset S9.

Supplementary Tables.

## Data Availability

The datasets generated and analysed during this study are available from the corresponding author upon reasonable request.
