# Supplementary material for: Diversity of voltage-gated potassium channels and cyclic nucleotide-binding domain-containing channels in eukaryotes
Source: Sci Rep. 2020 Oct 20;10:17758. doi: 10.1038/s41598-020-74971-4 (PMC7576140; doi:10.1038/s41598-020-74971-4)
Supplement: Supplementary file 10 — Supplementary Tables. [file 41598_2020_74971_MOESM10_ESM.pdf]

Supplementary Information

Diversity of voltage-gated potassium channels and cyclic nucleotide-binding domain-containing channels in eukaryotes

Ilya Pozdnyakov*, Pavel Safonov, Sergei Skarlato

Institute of Cytology, Russian Academy of Sciences, Saint Petersburg, 194064, Russia
***Corresponding author**: E-mail: pozdnyakov@incras.ru

№	Sequence name	Organism	Phylum	Accession number	Database
1	1x2Aph1292	*Aphanomyces invadans* (First repeat)	Oomycetes	XP_008861292.1	RefSeq
2	1x2Aph1293	*Aphanomyces invadans* (First repeat)	Oomycetes	XP_008861293.1	RefSeq
3	1x2Aph1311	*Aphanomyces invadans* (First repeat)	Oomycetes	XP_008861311.1	RefSeq
4	1x2Aph1313	*Aphanomyces invadans* (First repeat)	Oomycetes	XP_008861313.1	RefSeq
5	1x2Aph2798	*Aphanomyces invadans* (First repeat)	Oomycetes	XP_008862798.1	RefSeq
6	1x2Aph4712	*Aphanomyces invadans* (First repeat)	Oomycetes	XP_008874712.1	RefSeq
7	1x2Aph4713	*Aphanomyces invadans* (First repeat)	Oomycetes	XP_008874713.1	RefSeq
8	1x2At859	*Alexandrium tamarense CCMP1771* (First repeat)	Dinoflagellata	859_1	MMETSP
9	1x2Lot6	*Lotharella globosa CCCM811* (First repeat)	Chlorarachnea	116467_1	MMETSP
10	1x2Lot8	*Lotharella globosa CCCM811* (First repeat)	Chlorarachnea	81223_1	MMETSP
11	1x2Phyt7851	*Phytophthora infestans* (First repeat)	Oomycetes	XP_002907851.1	RefSeq

12	1x2Pm14899	*Prorocentrum minimum CCMP2233*<br>(First repeat)	Dinoflagellata	14899_1	MMETSP
13	1x2Pm156233	*Prorocentrum minimum CCMP2233*<br>(First repeat)	Dinoflagellata	156233_1	MMETSP
14	1x2Pm20439	*Prorocentrum minimum CCMP2233*<br>(First repeat)	Dinoflagellata	20439_1	MMETSP
15	1x2Pm255704	*Prorocentrum minimum CCMP2233*<br>(First repeat)	Dinoflagellata	255704_1	MMETSP
16	1x2Pm58510	*Prorocentrum minimum CCMP2233*<br>(First repeat)	Dinoflagellata	58510_1	MMETSP
17	1x2Pm906	*Prorocentrum minimum CCMP1329*<br>(First repeat)	Dinoflagellata	906_1	MMETSP
18	2x2Aph1292	*Aphanomyces invadans*<br>(Second repeat)	Oomycetes	XP_008861292.1	RefSeq
19	2x2Aph1293	*Aphanomyces invadans*<br>(Second repeat)	Oomycetes	XP_008861293.1	RefSeq
20	2x2Aph1311	*Aphanomyces invadans*<br>(Second repeat)	Oomycetes	XP_008861311.1	RefSeq
21	2x2Aph1313	*Aphanomyces invadans*<br>(Second repeat)	Oomycetes	XP_008861313.1	RefSeq

22	2x2Aph2798	*Aphanomyces invadans* (Second repeat)	Oomycetes	XP_008862798.1	RefSeq
23	2x2Aph4712	*Aphanomyces invadans* (Second repeat)	Oomycetes	XP_008874712.1	RefSeq
24	2x2Aph4713	*Aphanomyces invadans* (Second repeat)	Oomycetes	XP_008874713.1	RefSeq
25	2x2At859	*Alexandrium tamarense CCMP1771* (Second repeat)	Dinoflagellata	859_1	MMETSP
26	2x2Lot6	*Lotharella globosa CCCM811* (Second repeat)	Chlorarachnea	116467_1	MMETSP
27	2x2Lot8	*Lotharella globosa CCCM811* (Second repeat)	Chlorarachnea	81223_1	MMETSP
28	2x2Phyt7851	*Phytophthora infestans* (Second repeat)	Oomycetes	XP_002907851.1	RefSeq
29	2x2Pm14899	*Prorocentrum minimum CCMP2233* (Second repeat)	Dinoflagellata	14899_1	MMETSP
30	2x2Pm156233	*Prorocentrum minimum CCMP2233* (Second repeat)	Dinoflagellata	156233_1	MMETSP
31	2x2Pm20439	*Prorocentrum minimum CCMP2233* (Second repeat)	Dinoflagellata	20439_1	MMETSP
32	2x2Pm255704	*Prorocentrum minimum CCMP2233*	Dinoflagellata	255704_1	MMETSP

		(Second repeat)			
33	2x2Pm58510	*Prorocentrum minimum CCMP2233* (Second repeat)	Dinoflagellata	58510_1	MMETSP
34	2x2Pm906	*Prorocentrum minimum CCMP1329* (Second repeat)	Dinoflagellata	906_1	MMETSP
35	Acanth4xD1	*Acanthaster planci* (First repeat)	Metazoa	XP_022094112.1	RefSeq
36	Acanth4xD2	*Acanthaster planci* (Second repeat)	Metazoa	XP_022094112.1	RefSeq
37	Acanth4xD3	*Acanthaster planci* (Third repeat)	Metazoa	XP_022094112.1	RefSeq
38	Acanth4xD4	*Acanthaster planci* (Fourth repeat)	Metazoa	XP_022094112.1	RefSeq
39	Actinia4xD1	*Actinia tenebrosa* (First repeat)	Metazoa	XP_031557148.1	RefSeq
40	Actinia4xD2	*Actinia tenebrosa* (Second repeat)	Metazoa	XP_031557148.1	RefSeq
41	Actinia4xD3	*Actinia tenebrosa* (Third repeat)	Metazoa	XP_031557148.1	RefSeq
42	Actinia4xD4	*Actinia tenebrosa* (Fourth repeat)	Metazoa	XP_031557148.1	RefSeq
43	Aphanin1818	*Aphanomyces invadans*	Oomycetes	XP_008861818_1	RefSeq
44	AmaK	*Arthrospira maxima*	Bacteria	EDZ93391.1	GenBank
45	Aph0678	*Aphanomyces invadans*	Oomycetes	XP_008880678.1	RefSeq
46	Aph0917	*Aphanomyces invadans*	Oomycetes	XP_008880917.1	RefSeq
47	Aph1033	*Aphanomyces invadans*	Oomycetes	XP_008861033.1	RefSeq

48	Aph1039	*Aphanomyces invadans*	Oomycetes	XP_008861039.1	RefSeq
49	Aph2583	*Aphanomyces invadans*	Oomycetes	XP_008862583.1	RefSeq
50	Aph2584	*Aphanomyces invadans*	Oomycetes	XP_008862584.1	RefSeq
51	Aph3620	*Aphanomyces invadans*	Oomycetes	XP_008863620.1	RefSeq
52	Aph3634	*Aphanomyces invadans*	Oomycetes	XP_008863634.1	RefSeq
53	Aph4340	*Aphanomyces invadans*	Oomycetes	XP_008864340.1	RefSeq
54	Aph7182	*Aphanomyces invadans*	Oomycetes	XP_008867182.1	RefSeq
55	Aph8692	*Aphanomyces invadans*	Oomycetes	XP_008878692.1	RefSeq
56	Arabt0233	*Arabidopsis thaliana* (AKT1 channel)	Embryophyta	NP_180233.1	RefSeq
57	Arabt1280	*Arabidopsis thaliana* (SKOR channel)	Embryophyta	CAA11280.1	GenBank
58	Arabt4976	*Arabidopsis thaliana* (AKT5 channel)	Embryophyta	NP_194976.1	RefSeq
59	Arabt5440	*Arabidopsis thaliana* (GORK channel)	Embryophyta	OAO95440.1	GenBank
60	Arabt7380	*Arabidopsis thaliana* (GORK channel)	Embryophyta	CAC17380.1	GenBank
61	Arabt8922	*Arabidopsis thaliana* (AKT3 channel)	Embryophyta	NP_001328922.1	RefSeq
62	At102801	*Alexandrium tamarense CCMP1771*	Dinoflagellata	102801_1	MMETSP
63	At154789	*Alexandrium tamarense CCMP1771*	Dinoflagellata	154789_1	MMETSP

64	At157396	*Alexandrium tamarense CCMP1771*	Dinoflagellata	157396_1	MMETSP
65	At16160	*Alexandrium tamarense CCMP1771*	Dinoflagellata	16160_1	MMETSP
66	At165479	*Alexandrium tamarense CCMP1771*	Dinoflagellata	165479_1	MMETSP
67	At179206	*Alexandrium tamarense CCMP1771*	Dinoflagellata	179206_1	MMETSP
68	At18182	*Alexandrium tamarense CCMP1771*	Dinoflagellata	18182_1	MMETSP
69	At31712	*Alexandrium tamarense CCMP1771*	Dinoflagellata	31712_1	MMETSP
70	At35296	*Alexandrium tamarense CCMP1771*	Dinoflagellata	35296_1	MMETSP
71	At37804	*Alexandrium tamarense CCMP1771*	Dinoflagellata	37804_1	MMETSP
72	At39140	*Alexandrium tamarense CCMP1771*	Dinoflagellata	39140_1	MMETSP
73	At407021	*Alexandrium tamarense CCMP1771*	Dinoflagellata	407021_1	MMETSP
74	At414520	*Alexandrium tamarense CCMP1771*	Dinoflagellata	414520_1	MMETSP
75	At415146	*Alexandrium tamarense CCMP1771*	Dinoflagellata	415146_1	MMETSP
76	At51263	*Alexandrium tamarense CCMP1771*	Dinoflagellata	51263_1	MMETSP

77	At52477	*Alexandrium tamarense CCMP1771*	Dinoflagellata	52477_1	MMETSP
78	At5300	*Alexandrium tamarense CCMP1771*	Dinoflagellata	5300_1	MMETSP
79	At62880	*Alexandrium tamarense CCMP1771*	Dinoflagellata	62880_1	MMETSP
80	At73243	*Alexandrium tamarense CCMP1771*	Dinoflagellata	73243_1	MMETSP
81	At87079	*Alexandrium tamarense CCMP1771*	Dinoflagellata	87079_1	MMETSP
82	At980	*Alexandrium tamarense CCMP1771*	Dinoflagellata	980_1	MMETSP
83	Bodo1206	*Bodo saltans*	Kinetoplastea	CUG91206.1	GenBank
84	Bodo3028	*Bodo saltans*	Kinetoplastea	CUE73028.1	GenBank
85	Bodo5385	*Bodo saltans*	Kinetoplastea	CUG55385.1	GenBank
86	Bodo5404	*Bodo saltans*	Kinetoplastea	CUI15404.1	GenBank
87	Bodo6133	*Bodo saltans*	Kinetoplastea	CUF46133.1	GenBank
88	Bodo6702	*Bodo saltans*	Kinetoplastea	CUF66702.1	GenBank
89	Bodo7614	*Bodo saltans*	Kinetoplastea	CUG87614.1	GenBank
90	Bodo8773	*Bodo saltans*	Kinetoplastea	CUF38773.1	GenBank
91	Bodo8987	*Bodo saltans*	Kinetoplastea	CUF08987.1	GenBank
92	Bodo9837	*Bodo saltans*	Kinetoplastea	CUG89837.1	GenBank
93	Bodo9848	*Bodo saltans*	Kinetoplastea	CUG89848.1	GenBank
94	Bodo9929	*Bodo saltans*	Kinetoplastea	CUG89929.1	GenBank
95	Cel0829	*Caenorhabditis elegans* (SHK-1 channel)	Metazoa	NP_001040829.1	RefSeq
96	Cel3180	*Caenorhabditis elegans*	Metazoa	BAA13180.1	GenBank

97	Cel3402	*Caenorhabditis elegans*	Metazoa	NP_503402.3	RefSeq
98	Chaetneo1403	*Chaetoceros neogracile CCMP1317*	Bacillariophyta	1403_1	MMETSP
99	Chaetneo350	*Chaetoceros neogracile CCMP1317*	Bacillariophyta	350_1	MMETSP
100	Chaetneo3702	*Chaetoceros neogracile CCMP1317*	Bacillariophyta	13702_1	MMETSP
101	Chr13574	*Chrysochromulina polylepis CCMP1757*	Haptophyta	13574_1	MMETSP
102	Chr14044	*Chrysochromulina polylepis CCMP1757*	Haptophyta	14044_1	MMETSP
103	Chr15615	*Chrysochromulina polylepis CCMP1757*	Haptophyta	15615_1	MMETSP
104	Chr16314	*Chrysochromulina polylepis CCMP1757*	Haptophyta	16314_1	MMETSP
105	Chr16938	*Chrysochromulina polylepis CCMP1757*	Haptophyta	16938_1	MMETSP
106	Chr171091	*Chrysochromulina polylepis CCMP1757*	Haptophyta	171091_1	MMETSP
107	Chr172295	*Chrysochromulina polylepis CCMP1757*	Haptophyta	172295_1	MMETSP
108	Chr172507	*Chrysochromulina polylepis CCMP1757*	Haptophyta	172507_1	MMETSP
109	Chr173054	*Chrysochromulina polylepis CCMP1757*	Haptophyta	173054_1	MMETSP

110	Chr173105	*Chrysochromulina polylepis CCMP1757*	Haptophyta	173105_1	MMETSP
111	Chr21728	*Chrysochromulina polylepis CCMP1757*	Haptophyta	21728_1	MMETSP
112	Chr22465	*Chrysochromulina polylepis CCMP1757*	Haptophyta	22465_1	MMETSP
113	Chr25304	*Chrysochromulina polylepis CCMP1757*	Haptophyta	25304_1	MMETSP
114	Chr27069	*Chrysochromulina polylepis CCMP1757*	Haptophyta	27069_1	MMETSP
115	Chr3027	*Chrysochromulina polylepis CCMP1757*	Haptophyta	3027_1	MMETSP
116	Chr40677	*Chrysochromulina polylepis CCMP1757*	Haptophyta	40677_1	MMETSP
117	Chr49074	*Chrysochromulina polylepis CCMP1757*	Haptophyta	49074_1	MMETSP
118	Chr6780	*Chrysochromulina polylepis CCMP1757*	Haptophyta	6780_1	MMETSP
119	Chr6793	*Chrysochromulina polylepis CCMP1757*	Haptophyta	6793_1	MMETSP
120	Chr8707	*Chrysochromulina polylepis CCMP1757*	Haptophyta	18707_1	MMETSP
121	Chr9301	*Chrysochromulina polylepis CCMP1757*	Haptophyta	9301_1	MMETSP
122	Cryptog4458	*Cryptococcus gattii*	Fungi	KIR84458.1	GenBank
123	Danio4xD1	*Danio rerio* (First repeat)	Metazoa	XP_001335499.5	RefSeq

124	Danio4xD2	*Danio rerio* (Second repeat)	Metazoa	XP_001335499.5	RefSeq
125	Danio4xD3	*Danio rerio* (Third repeat)	Metazoa	XP_001335499.5	RefSeq
126	Danio4xD4	*Danio rerio* (Fourth repeat)	Metazoa	XP_001335499.5	RefSeq
127	Dros122	*Drosophila melanogaster* (Shaker)	Metazoa	NP_728122.1	RefSeq
128	Dros4470	*Drosophila melanogaster*	Metazoa	ACV44470.1	GenBank
129	Dros7256	*Drosophila busckii* (HCN2 channel)	Metazoa	XP_017837256.1	RefSeq
130	Dros8960	*Drosophila melanogaster* (CNG channel)	Metazoa	NP_001188960_1	RefSeq
131	Emilih1782	*Emiliania huxleyi CCMP370*	Haptophyta	21782_1	MMETSP
132	Emilih183686	*Emiliania huxleyi CCMP370*	Haptophyta	183686_1	MMETSP
133	Emilih21357	*Emiliania huxleyi CCMP370*	Haptophyta	21357_1	MMETSP
134	Emilih5070	*Emiliania huxleyi CCMP370*	Haptophyta	35070_1	MMETSP
135	Goniopac0043	*Goniomonas pacifica CCMP1869*	Cryptophyta	30043_1	MMETSP
136	Goniopac1159	*Goniomonas pacifica CCMP1869*	Cryptophyta	31159_1	MMETSP
137	Goniopac2926	*Goniomonas pacifica CCMP1869*	Cryptophyta	2926_1	MMETSP
138	Goniopac360812	*Goniomonas pacifica CCMP1869*	Cryptophyta	360812_1	MMETSP
139	Goniopac4299	*Goniomonas pacifica CCMP1869*	Cryptophyta	234299_1	MMETSP
140	Goniopac4884	*Goniomonas pacifica CCMP1869*	Cryptophyta	34884_1	MMETSP

141	Hond1530	*Hondaea fermentalgiana*	Labyrinthulomycetes	GBG31530.1	GenBank
142	Hond3016	*Hondaea fermentalgiana*	Labyrinthulomycetes	GBG33016.1	GenBank
143	Hond4246	*Hondaea fermentalgiana*	Labyrinthulomycetes	GBG34246.1	GenBank
144	Hond5239	*Hondaea fermentalgiana*	Labyrinthulomycetes	GBG25239.1	GenBank
145	Hond7007	*Hondaea fermentalgiana*	Labyrinthulomycetes	GBG27007.1	GenBank
146	Hond7128	*Hondaea fermentalgiana*	Labyrinthulomycetes	GBG27128.1	GenBank
147	Hond8904	*Hondaea fermentalgiana*	Labyrinthulomycetes	GBG28904.1	GenBank
148	Hond9142	*Hondaea fermentalgiana*	Labyrinthulomycetes	GBG29142.1	GenBank
149	Hond9401	*Hondaea fermentalgiana*	Labyrinthulomycetes	GBG29401.1	GenBank
150	Hs1185	*Homo sapiens* (HCN2 channel)	Metazoa	NP_001185.3	RefSeq
151	Hs6036	*Homo sapiens* (CNG1 channel)	Metazoa	NP_001136036.1	RefSeq
152	HsEAGREF	*Homo sapiens* (EAG1 channel)	Metazoa	NP_758872.1	RefSeq
153	HsHCNREF	*Homo sapiens* (HCN1 channal)	Metazoa	NP_066550.2	RefSeq
154	HsKvREF	*Homo sapiens* (Kv1.7 channel)	Metazoa	AAX11186.1	GenBank
155	KvAP	Aeropyrum pernix K1	Archaea	Q9YDF8.1	GenBank
156	KvLm	Listeria monocytogenes EGD-e	Bacteria	NP_465583.1	RefSeq
157	LbiK	*Leptospira biflexa*	Bacteria	ABZ94327.1	GenBank
158	Lingula4xD1	*Lingula anatine*	Metazoa	XP_013410596.1	RefSeq

		First domain			
159	Lingula4xD2	*Lingula anatine* Second domain	Metazoa	XP_013410596.1	RefSeq
160	Lingula4xD3	*Lingula anatine* Third domain	Metazoa	XP_013410596.1	RefSeq
161	Lingula4xD4	*Lingula anatine* Fourth domain	Metazoa	XP_013410596.1	RefSeq
162	Lot0433	*Lotharella globosa CCCM811*	Chlorarachnea	120433_1	MMETSP
163	Lot80703	*Lotharella globosa CCCM811*	Chlorarachnea	80703_1	MMETSP
164	Lot85122	*Lotharella globosa CCCM811*	Chlorarachnea	85122_1	MMETSP
165	Lot86777	*Lotharella globosa CCCM811*	Chlorarachnea	86777_1	MMETSP
166	Lot9618	*Lotharella globosa CCCM811*	Chlorarachnea	9618_1	MMETSP
167	Lottia3678	*Lottia gigantea*	Metazoa	XP_009063678.1	RefSeq
168	Lottia4816	*Lottia gigantea*	Metazoa	XP_009054816.1	RefSeq
169	Lottia5598	*Lottia gigantea*	Metazoa	XP_009055598.1	RefSeq
170	Lottia5762	*Lottia gigantea*	Metazoa	XP_009045762.1	RefSeq
171	LpcK	*Lyngbya sp.*	Bacteria	WP_009782512.1	RefSeq
172	Mneml0466	*Mnemiopsis leidyi*	Metazoa	AIQ80466.1	GenBank
173	Mneml3742	*Mnemiopsis leidyi*	Metazoa	AGB13742.1	GenBank
174	Mneml9346	*Mnemiopsis leidyi*	Metazoa	AJP09346.1	GenBank
175	Monbr6676	*Monosiga brevicollis*	Choanoflagellata	XP_001746676.1	RefSeq
176	Monbr9885	*Monosiga brevicollis*	Choanoflagellata	XP_001749885.1	RefSeq
177	Mortierel0503	*Mortierella elongata*	Fungi	OAQ30503.1	GenBank
178	Mortierel9832	*Mortierella elongata*	Fungi	OAQ29832.1	GenBank
179	MVP	Methanocaldococcus jannaschii DSM 2661	Archaea	Q57603	GenBank

180	Orbicf0666	*Orbicella faveolata*	Metazoa	XP_020610666.1	RefSeq
181	Orbicf5061	*Orbicella faveolata*	Metazoa	XP_020605061.1	RefSeq
182	Orbicf8378	*Orbicella faveolata*	Metazoa	XP_020608378.1	RefSeq
183	Orbicf8450	*Orbicella faveolata*	Metazoa	XP_020618450.1	RefSeq
184	Ostr0014	*Ostreococcus tauri*	Chlorophyta	CEG00014.1	GenBank
185	Ostr0029	*Ostreococcus tauri*	Chlorophyta	CEG00029.1	GenBank
186	Ostr0041	*Ostreococcus tauri*	Chlorophyta	CEG00041.1	GenBank
187	Ostr2923	*Ostreococcus tauri*	Chlorophyta	OUS42923.1	GenBank
188	Ostr3585	*Ostreococcus tauri*	Chlorophyta	OUS43585.1	GenBank
189	Ostr4104	*Ostreococcus tauri*	Chlorophyta	XP_003074104.1	RefSeq
190	Ostr6137	*Ostreococcus tauri*	Chlorophyta	OUS46137.1	GenBank
191	Ostr6492	*Ostreococcus tauri*	Chlorophyta	CEF96492.1	GenBank
192	Ostr7909	*Ostreococcus tauri*	Chlorophyta	CEF97909.1	GenBank
193	Part0078	*Paramecium tetraurelia*	Ciliophora	XP_001440078.1	RefSeq
194	Part1576	*Paramecium tetraurelia*	Ciliophora	XP_001451576.1	RefSeq
195	Part1680	*Paramecium tetraurelia*	Ciliophora	XP_001431680.1	RefSeq
196	Part2060	*Paramecium tetraurelia*	Ciliophora	XP_001452060.1	RefSeq
197	Part2164	*Paramecium tetraurelia*	Ciliophora	XP_001452164.1	RefSeq
198	Part3746	*Paramecium tetraurelia*	Ciliophora	XP_001443746.1	RefSeq
199	Part3991	*Paramecium tetraurelia*	Ciliophora	XP_001423991.1	RefSeq
200	Part4007	*Paramecium tetraurelia*	Ciliophora	XP_001454007.1	RefSeq
201	Part5168	*Paramecium tetraurelia*	Ciliophora	XP_001455168.1	RefSeq
202	Part5598	*Paramecium tetraurelia*	Ciliophora	XP_001445598.1	RefSeq
203	Part5644	*Paramecium tetraurelia*	Ciliophora	XP_001425644.1	RefSeq

204	Part6349	*Paramecium tetraurelia*	Ciliophora	XP_001426349.1	RefSeq
205	Part7227	*Paramecium tetraurelia*	Ciliophora	XP_001347227.1	RefSeq
206	Part7798	*Paramecium tetraurelia*	Ciliophora	XP_001437798.1	RefSeq
207	Part7972	*Paramecium tetraurelia*	Ciliophora	XP_001457972.1	RefSeq
208	Part8397	*Paramecium tetraurelia*	Ciliophora	XP_001438397.1	RefSeq
209	Part8423	*Paramecium tetraurelia*	Ciliophora	XP_001448423.1	RefSeq
210	Part8681	*Paramecium tetraurelia*	Ciliophora	XP_001438681.1	RefSeq
211	Part8783	*Paramecium tetraurelia*	Ciliophora	XP_001448783.1	RefSeq
212	Part8867	*Paramecium tetraurelia*	Ciliophora	XP_001458867.1	RefSeq
213	Part9500	*Paramecium tetraurelia*	Ciliophora	XP_001429500.1	RefSeq
214	Part9850	*Paramecium tetraurelia*	Ciliophora	XP_001459850.1	RefSeq
215	Perkm7058	*Perkinsus marinus*	Perkinsidae	XP_002767058.1	RefSeq
216	Perkm8060	*Perkinsus marinus*	Perkinsidae	XP_002768060.1	RefSeq
217	Phyt5745	*Phytophthora infestans*	Oomycetes	XP_002895745.1	RefSeq
218	Pm100515	*Prorocentrum minimum CCMP2233*	Dinoflagellata	100515_1	MMETSP
219	Pm11858	*Prorocentrum minimum CCMP2233*	Dinoflagellata	11858_1	MMETSP
220	Pm124306	*Prorocentrum minimum CCMP2233*	Dinoflagellata	124306_1	MMETSP
221	Pm13328	*Prorocentrum minimum CCMP2233*	Dinoflagellata	13328_1	MMETSP

222	Pm14163	*Prorocentrum minimum CCMP2233*	Dinoflagellata	14163_1	MMETSP
223	Pm255449	*Prorocentrum minimum CCMP2233*	Dinoflagellata	255449_1	MMETSP
224	Pm256265	*Prorocentrum minimum CCMP2233*	Dinoflagellata	256265_1	MMETSP
225	Pm258592	*Prorocentrum minimum CCMP2233*	Dinoflagellata	258592_1	MMETSP
226	Pm260406	*Prorocentrum minimum CCMP2233*	Dinoflagellata	260406_1	MMETSP
227	Pm30427	*Prorocentrum minimum CCMP2233*	Dinoflagellata	30427_1	MMETSP
228	Pm31544	*Prorocentrum minimum CCMP2233*	Dinoflagellata	31544_1	MMETSP
229	Pm35067	*Prorocentrum minimum CCMP2233*	Dinoflagellata	35067_1	MMETSP
230	Pm35426	*Prorocentrum minimum CCMP2233*	Dinoflagellata	35426_1	MMETSP
231	Pm44058	*Prorocentrum minimum CCMP2233*	Dinoflagellata	44058_1	MMETSP
232	Pm8705	*Prorocentrum minimum CCMP2233*	Dinoflagellata	8705_1	MMETSP
233	Rhiz1414	*Rhizophagus irregularis*	Fungi	EXX51414.1	GenBank
234	Rhiz1575	*Rhizophagus irregularis*	Fungi	EXX51575.1	GenBank
235	Rhiz7733	*Rhizophagus irregularis*	Fungi	EXX77733.1	GenBank

236	Rhodm103319	*Rhodella maculata CCMP736*	Rhodophyceae	103319_1	MMETSP
237	Rhodm14260	*Rhodella maculata CCMP736*	Rhodophyceae	14260_1	MMETSP
238	Rhodm50364	*Rhodella maculata CCMP736*	Rhodophyceae	50364_1	MMETSP
239	Rhodm9830	*Rhodella maculata CCMP736*	Rhodophyceae	9830_1	MMETSP
240	Rhodomsp3660	*Rhodomonas sp. CCMP768*	Cryptophyta	13660_1	MMETSP
241	Rhodomsp4159	*Rhodomonas sp. CCMP768*	Cryptophyta	4159_1	MMETSP
242	Rhodomsp4271	*Rhodomonas sp. CCMP768*	Cryptophyta	134271_1	MMETSP
243	Rhodomsp4926	*Rhodomonas sp. CCMP768*	Cryptophyta	134926_1	MMETSP
244	Rhodomsp5962	*Rhodomonas sp. CCMP768*	Cryptophyta	15962_1	MMETSP
245	Rhodomsp6827	*Rhodomonas sp. CCMP768*	Cryptophyta	16827_1	MMETSP
246	Saitoella2107	*Saitoella complicata*	Fungi	XP_019022107.1	RefSeq
247	Salp4xD1	*Salpingoeca rosetta* (First repeat)	Choanoflagellata	XP_004992545.1	RefSeq
248	Salp4xD2	*Salpingoeca rosetta* (Second repeat)	Choanoflagellata	XP_004992545.1	RefSeq
249	Salp4xD3	*Salpingoeca rosetta* (Third repeat)	Choanoflagellata	XP_004992545.1	RefSeq
250	Salp4xD4	*Salpingoeca rosetta* (Fourth repeat)	Choanoflagellata	XP_004992545.1	RefSeq
251	Salp1538	*Salpingoeca rosetta*	Choanoflagellata	XP_004991538.1	RefSeq
252	SthK	*Spirochaeta thermophila*	Bacteria	WP_013313430.1	RefSeq
253	Str0120	*Stentor coeruleus*	Ciliophora	OMJ80120.1	GenBank
254	Str0956	*Stentor coeruleus*	Ciliophora	OMJ 70956.1	GenBank
255	Str1302	*Stentor coeruleus*	Ciliophora	OMJ 71302.1	GenBank

256	Str1316	*Stentor coeruleus*	Ciliophora	OMJ 71316.1	GenBank
257	Str1416	*Stentor coeruleus*	Ciliophora	OMJ 81416.1	GenBank
258	Str1659	*Stentor coeruleus*	Ciliophora	OMJ 91659.1	GenBank
259	Str2484	*Stentor coeruleus*	Ciliophora	OMJ 72484.1	GenBank
260	Str2486	*Stentor coeruleus*	Ciliophora	OMJ 72486.1	GenBank
261	Str2853	*Stentor coeruleus*	Ciliophora	OMJ 72853.1	GenBank
262	Str2857	*Stentor coeruleus*	Ciliophora	OMJ 92857.1	GenBank
263	Str3241	*Stentor coeruleus*	Ciliophora	OMJ 73241.1	GenBank
264	Str3433	*Stentor coeruleus*	Ciliophora	OMJ 73433.1	GenBank
265	Str3516	*Stentor coeruleus*	Ciliophora	OMJ 93516.1	GenBank
266	Str3541	*Stentor coeruleus*	Ciliophora	OMJ 93541.1	GenBank
267	Str4014	*Stentor coeruleus*	Ciliophora	OMJ 84014.1	GenBank
268	Str4420	*Stentor coeruleus*	Ciliophora	OMJ 94420.1	GenBank
269	Str4679	*Stentor coeruleus*	Ciliophora	OMJ 74679.1	GenBank
270	Str5032	*Stentor coeruleus*	Ciliophora	OMJ 85032.1	GenBank
271	Str5197	*Stentor coeruleus*	Ciliophora	OMJ 95197.1	GenBank
272	Str5952	*Stentor coeruleus*	Ciliophora	OMJ 95952.1	GenBank
273	Str5999	*Stentor coeruleus*	Ciliophora	OMJ 75999.1	GenBank
274	Str6016	*Stentor coeruleus*	Ciliophora	OMJ 86016.1	GenBank
275	Str6145	*Stentor coeruleus*	Ciliophora	OMJ 86145.1	GenBank
276	Str6239	*Stentor coeruleus*	Ciliophora	OMJ 76239.1	GenBank
277	Str6256	*Stentor coeruleus*	Ciliophora	OMJ 86256.1	GenBank
278	Str6710	*Stentor coeruleus*	Ciliophora	OMJ 66710.1	GenBank
279	Str7755	*Stentor coeruleus*	Ciliophora	OMJ 77755.1	GenBank
280	Str7834	*Stentor coeruleus*	Ciliophora	OMJ 87834.1	GenBank
281	TerK	*Trichodesmium erythraeum*	Bacteria	WP_011614153.1	RefSeq
282	Tfet5153	*Tritrichomonas foetus*	Trichomonadida	OHT15153.1	GenBank
283	Thalassm834790	*Thalassiosira miniscula CCMP1093*	Bacillariophyta	834790_1	MMETSP

284	Thecat1176	*Thecamonas trahens*	Apusomonadida	XP_013761176.1	RefSeq
285	Thecat1364	*Thecamonas trahens*	Apusomonadida	XP_013761364.1	RefSeq
286	Thecat4561	*Thecamonas trahens*	Apusomonadida	XP_013754561.1	RefSeq
287	Thecat5195	*Thecamonas trahens*	Apusomonadida	XP_013755195.1	RefSeq
288	Thecat6466	*Thecamonas trahens*	Apusomonadida	XP_013756466.1	RefSeq
289	Thecat8941	*Thecamonas trahens*	Apusomonadida	XP_013758941.1	RefSeq
290	Thecat9688	*Thecamonas trahens*	Apusomonadida	XP_013759688.1	RefSeq
291	Thecat9915	*Thecamonas trahens*	Apusomonadida	XP_013759915.1	RefSeq
292	Tvag6965	*Trichomonas vaginalis G3*	Trichomonadida	XP_001326965.1	RefSeq
293	TrichKv	*Trichoplax sp. H2*	Metazoa	RDD43142.1	GenBank
294	Vitr5198	*Vitrella brassicaformis*	Colpodellida	CEM05198.1	GenBank
295	Vitr8094	*Vitrella brassicaformis*	Colpodellida	CEM28094.1	GenBank
296	Vitr9089	*Vitrella brassicaformis*	Colpodellida	CEL99089.1	GenBank
297	Volvox0072	*Volvox carteri f. nagariensis*	Chlorophyta	XP_002950072.1	GenBank
298	Volvox2111	*Volvox carteri f. nagariensis*	Chlorophyta	XP_002952111.1	GenBank
299	Volvox2132	*Volvox carteri f. nagariensis*	Chlorophyta	XP_002952132.1	GenBank
300	Volvox5751	*Volvox carteri f. nagariensis*	Chlorophyta	XP_002945751.1	GenBank
301	Volvox8978	*Volvox carteri f. nagariensis*	Chlorophyta	XP_002948978.1	GenBank
302	Volvox9510	*Volvox carteri f. nagariensis*	Chlorophyta	XP_002959510.1	GenBank

Table S1. List of amino acid sequences of K_V-like channels (yellow) and CNBD-channels (blue) used in the research. The repeats of tandem channels are listed as separate sequences.

№	Sequence name	Linker S4-S5	S4	SF	CNBD
1	1x2Aph1292	------IVLR----TFLEC	RRQIRYMKLLRLFRIIRVYMLRDVD-	TVGYG	Abs.
2	1x2Aph1293	------IVLR----TFLEC	RRQIRYMKLLRLFRIIRVYMLRDVD-	TVGYG	Abs.
3	1x2Aph1311	------IVLH----AFLEC	RYAIEYLRHLRLFRIVRVYMLRNMS-	TVGYG	Abs.
4	1x2Aph1313	------MILY----YAKES	IQLSQYIKLLRLFRIIRVTMLKNTS-	TVGYG	Abs.
5	1x2Aph2798	GTY---ILFQ----AIRAS	-----SFVVMRLARLLRIFRLAKNNS	TVGYG	Abs.
6	1x2Aph4712	---FAN---Q	AKILRGLKLIRLLKLVRIRKIGQMIS	TVGYG	Pres.
7	1x2Aph4713	---FAN---Q	AKIFRGLKLVRLLKLVRIRKIGNMIS	TIGYG	Pres.
8	1x2At859	GMQ---LIAR----VMLRS	--TLGFLRVLRLARVLRILKLAKRNQ	TVGYG	Abs.
9	1x2Lot6	MIP---LLVT----VLERS	SNVLTSLRIARLVRLAKLFKRNRM--	TVGYG	Abs.
10	1x2Lot8	MIP---LMLK----VVGRA	AELLAPLRIVRLLRLVRLFKGNTM--	TVGYG	Abs.
11	1x2Phyt7851	-----NA---	LKLIRVFRLFRLLKLMRLLRLKRTME	SVGYG	Pres.
12	1x2Pm14899	---NLN---P	IKF---VRLVRLVKLMRLLKLSRFVR	AVGYG	Red.
13	1x2Pm156233	ETK---VFMD----TMIMS	RSAKSIVSVFRLGRILRLFKVTKYLP	TIGYG	Abs.
14	1x2Pm20439	GMK---MFAK----VLYKS	---LGSLEILRLARILRIFKMARHHP	TVGYG	Abs.
15	1x2Pm255704	GVK---IFWE----VIVMS	--GDSKIAVLRLARILRIFKLAKHHP	TVGYG	Abs.
16	1x2Pm58510	---------AM	LRDFRVMRLLRLAKLARLGRLRRLTE	AVGYG	Pres.
17	1x2Pm906	GMK---MFAK----VLYKS	---LGSLEILRLARILRIFKMARHHP	TVGYG	Abs.
18	2x2Aph1292	ASS---VLAR----TAVLT	--TFPTMSVLPILKTLRVLKMGKHFK	TVGYG	Abs.
19	2x2Aph1293	ASS---VLAR----TAVLT	--TFPTMSVLPILKTLRVLKMGKHFK	TVGYG	Abs.
20	2x2Aph1311	GSS---VLAR----TAALT	--TFPTLSILPIMKTLRIVKLGWYLQ	GVGYG	Abs.
21	2x2Aph1313	STM---VLVR----TAELT	--TFPTISVVPMTKTLRIFKLAKHFK	TVGYG	Abs.
22	2x2Aph2798	VVS----------------	--EMDILSTLRFARIFRWFQI----K	SVGYG	Abs.
23	2x2Aph4712	---QLS---P	LRM---FRVARLFKLVRLLKIGKVFK	TVGYG	Pres.
24	2x2Aph4713	---QLS---P	LRV---FRVARLFKLVRLLKLGKVFK	TVGYG	Pres.
25	2x2At859	GVT---MFAN----IVADS	GAGTSVLRVFRLVRIFRMLRMPKLSS	TVGYG	Abs.
26	2x2Lot6	GLR---IMAA----AIDNS	--SLTFFRILRLLRMMRILRLAGSLK	TVGYG	Abs.
27	2x2Lot8	AVV---ILSD----AIKKS	--SLEILRVVRLLRIARVLRLARKFK	TVGFG	Abs.
28	2x2Phyt7851	---DIN---V	IRT---LKLIKLLRLMRLSKLLKMNS	TVGFG	Pres.
29	2x2Pm14899	---EVS---P	ARALKMFKTLRLLRLLKLARLMRLVR	TVGYG	Pres.
30	2x2Pm156233	GVM---MLMN----VVVDC	--GLAVLRVLRLVRTLRVLKMPKLRL	TVGYG	Abs.
31	2x2Pm20439	GVA---MFSH----VIMDS	GGGMSAIRVVRLARIFRVLRMRKLQT	TVGYG	Abs.
32	2x2Pm255704	CVA---MFGQ----IITDS	--ATSVLRILRLVRVFRVLRMPQLSS	TVGYG	Abs.
33	2x2Pm58510	---DVS---T	TAASRLLRFTRLTRMLKMARVIARLS	TVGYG	Red.
34	2x2Pm906	GVA---MFSH----VIMDS	GGGMSAIRVVRLARIFRVLRMRKLQT	TVGYG	Abs.
35	Acanth4xD1	-KLGSN---T	MKVLAKY--RCMNRWIRCFRLMSFFE	STGYG	Pres.
36	Acanth4xD2	-DLDVN---I	TNALYF---LSLNRLVKAWRMPNYFK	STGYG	Pres.
37	Acanth4xD3	-GVENE---T	IHVYAV---LRLNRFLQMYRVPLAFN	SVGYG	Pres.
38	Acanth4xD4	-ELNIN---M	FAALTL---LRIVHLLRLVRVVQFFD	STGYG	Pres.
39	Actinia4xD1	-ELGSN---T	--------------------MMKFFD	STGYG	Pres.
40	Actinia4xD2	-RLEYN---I	WHAVTF---LRLNRLLKVLRMSAFFS	STGYG	Pres.
41	Actinia4xD3	-RLGKS---E	LRILAF---ARLNRCLHIYKIALFFG	SVGYG	Pres.
42	Actinia4xD4	-KLDID---V	LEILSF---LRLLHLLRLVRMEQFFT	STGYG	NI
43	Aphanin1818	---------R	VKLEKLPKLLRFGILLKYLRQ-----	GIGEG	Pres.
44	AmaK	---KIN---P	--SVLILAILRIPRLLRLSQFFRIFR	TVGYG	Pres.
45	Aph0678	---SIN---Y	LRFTRVLRLLRLMKILKVLRGASNFR	TIGYG	Pres.

46	Aph0917	AAT---LMDN----IHQN-	--HLLVLRILRVFRILKLVRFMEAA-	TVGYG	Abs.
47	Aph1033	SIQ---IFIK----ALILS	GSSIGAIRIMRLIRVARILKLSRYTS	TVGYG	Abs.
48	Aph1039	SLQ---IFVK----SLSLS	ASSLSAIRTMRLLRVARILKLSKYTS	TVGYG	Abs.
49	Aph2583	---LIK---S	ARLNKYFKFLRIIKLTRMLGFTHILN	TVGYG	Pres.
50	Aph2584	---LIK---S	ARLNKYFKFLRIIKLTRMLGFTHILN	TVGYG	Red.
51	Aph3620	---NIP---M	FRI---LRILRIIKLVRVFRASRIFS	TIGYG	Pres.
52	Aph3634	---GFK---Y	IRI---IRLFRLIKLVRVFRASRIYS	TVGYG	Pres.
53	Aph4340	---LIR---S	--LGKVLRLFRLVRLLRLFRMIRILN	TVGYG	Pres.
54	Aph7182	---GLP---I	IRV------FRILKLARVFRASRILA	TIGYG	Pres.
55	Aph8692	---SIK---Y	LRLPRLLRMFRLTKILKV---SRIFQ	TIGYG	Pres.
56	Arabt0233	---NFN---Y	MRISQ-SYLFNMLRLWRLRRVGALFA	TVGYG	Pres.
57	Arabt1280	---RIN---Y	VR-----YLL-LIRLYRVHRVILFFH	TVGYG	Pres.
58	Arabt4976	---KYN---Y	LHNIQ-GYIFSMLRLWRLHRVSKCFA	TTGYG	Pres.
59	Arabt5440	---RIN---Y	VR-----YLL-WIRLFRVRKVVEFFQ	TVGYG	Pres.
60	Arabt7380	---RIN---Y	VR-----YLL-WIRLFRVRKVVEFFQ	TVGYG	Pres.
61	Arabt8922	---RIN---Y	VR-----YLL-WIRLFRVRKVVEFFQ	TVGYG	Pres.
62	At102801	---NLQ---F	AKILRCIKLMRLTKLLRILRSSKIME	SIGYG	Red.
63	At154789	--------NS	LRALRILRLFRLVRLFRLSRLFRYLG	CIGYG	Pres.
64	At157396	TIS---TVFE----SIWDS	MRTLRMVRLIRMLRVLRLAKAARHSE	TVGYG	Abs.
65	At16160	---DLP---Y	LRL---VRLLRLIKLARVLRASRLFK	PMGVG	Pres.
66	At165479	---NQA--FM	IRA---FRLLRLTRLVRLLRSNALTE	TVGYG	Pres.
67	At179206	AVV---IIAK----AMHRS	DSTLVLLKLTRLMRVVRAFRLGRRFE	TVGYG	Abs.
68	At18182	GLQ---LMIL----AMRNS	TKALGVLRTVRLVRLFRIFKLGRYSV	TVGFG	Abs.
69	At31712	VVA---TVLQ----SFYAS	QKTVAMLRILQIMRIVRIAKAARHSE	GVGYG	Abs.
70	At35296	---KQS--LC	MMLARSLRLTRLARLAGVLKANKLVA	TVGYG	Pres.
71	At37804	PVI---VISQ----TISQS	F---VVLRLIRLTRIFRAFKNPALAE	TVGYG	Abs.
72	At39140	AVI---IIAR----SVRRS	E---FILKILRLTRVIRAFRLGRRFE	TVGYG	Abs.
73	At407021	A-AQSM---T	VGMLKVLRYAKVARMMKVLRVLKLGG	TVGYG	Pres.
74	At414520	---SIN---R	LRVVKAFRVLRLTKLVRFLKLSAVIE	TVGYG	Pres.
75	At415146	---SIN---P	FRVTKLLKMVRMVRLLKLTRFLELFV	TVGYG	Pres.
76	At51263	---DMS---Y	LRT---IRLLRLIKLARVFRASRLFK	PMGVG	Pres.
77	At52477	---GFS---Y	LEI------IRLIRLLRLTRLARLFG	SIGYG	Pres.
78	At5300	---GIS---N	LRY------FRAVRLLRLVRFVRLVG	SIGYG	Pres.
79	At62880	VLE---IIVR----VFRRS	ASNFTVLRVLRLTRLFRLIKLGKYFE	TVGYG	Abs.
80	At73243	GMN---LMVE----SLVNS	LKAFRVLRAARLIRVFRIFKLSKYSL	TVGYG	Abs.
81	At87079	AVI---IIAR----SMHRS	DSSMVILKLIRLMRVVRAFRLGRRFE	TAGYG	Abs.
82	At980	A-AQSM---T	MGMLKVLRYAKVARMMKVLRVLKLGS	TVGYG	Pres.
83	Bodo1206	GMQ---VWG-----SVVKS	--ILILIRLLRLTRILRVFDLSKHNV	TTGYG	Abs.
84	Bodo3028	PVR---MLQK----ALKFA	--ITGTLRTVRLLRLFKILRFYKPVR	TTGFG	Abs.
85	Bodo5385	GLA---MLWQ----VLLKS	SGGLALLRLLRIARILRLFKLGRQDS	TVGYG	Abs.
86	Bodo5404	------LVETL---RIASS	--WLKTLRLFRLVKLLRMFQF-----	TTGYG	Abs.
87	Bodo6133	------ELFR----AMRKS	PDGLAVIRVIRLIRTVRLMRS----S	TVGYG	Abs.
88	Bodo6702	ILG---KLLN----TVTSS	--FLRIIRALRIARLVKVLKS----R	TAGIG	Abs.
89	Bodo7614	---------K	ISMLKILAHFRIVKLQRQIQITDHYV	TAGYG	Pres.
90	Bodo8773	GMN---IME-----SVRLS	--DLRVFFVIRMSRVIRL---SRYHK	LNGFG	Abs.
91	Bodo8987	------FLVQV---TVVGS	--YVSMLRAIRVFRFVYLFKD----Y	LTGYG	Abs.
92	Bodo9837	------MLLH----TLERS	LAPLKVLRVLRLLRALRVLHS-----	NVGYG	Abs.

93	Bodo9848	---TRN---F	--LIDIMRLLKIFMVLRCVKYSRLF-	TVGYG	Pres.
94	Bodo9929	SLQ---LVVI----VLRKS	AGGVAVVRLLRLFRVFRVFKLSRHSK	TVGFG	Abs.
95	Cel0829	GLQ---ILGK----TFRSS	SMSFAVLRVLRLVRVFRVFKLSRHSV	TVGYG	Abs.
96	Cel3180	-------SMP	-----GLPILRLNRLIRYKRVRNCLE	TI--G	Pres.
97	Cel3402	---E-----Y	GSL---FSALKVVRLLRLGRVARKLD	TVGFG	Pres.
98	Chaetneo1403	RFQ---KALG----MKRNT	--GVQNLLLLRILRIRRVLTDINTFG	TVGFG	Abs.
99	Chaetneo350	KFE---LALG----LNPSA	LDDLINLRLLRILRLQRVLIDYETFT	TVGFG	Abs.
100	Chaetneo3702	MMV----------------	------LRYIRLLRFFRVIRL----D	TAGYG	Abs.
101	Chr13574	GVQ---MMAA----TMMES	VGAFSVFRVIRLVRVFRIFKMGKASG	TVGYG	Abs.
102	Chr14044	---AIN---Y	FKVLRVLRVLRLIKLVRLVRASRIAK	SIGYG	Pres.
103	Chr15615	GL----VLAT----TFRKS	LDVLSVLRLIRLTRITRIFKMSKNFQ	TVGYG	Abs.
104	Chr16314	GIK---VFAG----ALRLS	TS---FLRAVRLFRIFKIFKSGQYSV	TVGYG	Abs.
105	Chr16938	-ELDLN---M	LRMLRMIRILRFMKLIRLMKAAKIFR	TIGYG	Pres.
106	Chr171091	-------STK	GRLTRLIRTIKMIRLLRLLKLIRYFA	SIGYG	Pres.
107	Chr172295	--------SG	--ALRALQLVKLARLMRLKRLAKLN-	SLGLG	Pres.
108	Chr172507	RIE---VLSS----LLR--	LSLLPILRLVRLLRLFRLFKLSRFVK	TVGYG	Abs.
109	Chr173054	GLI---VLGN----TIMKS	LGFLQILRLIRLTRIVRIFKMSKNFE	TVGYG	Abs.
110	Chr173105	---QVN---P	AKLLKGLRLVRLMKLLRLLKITKYLH	TIGYG	Pres.
111	Chr21728	GLQ---LFGR----TLLSS	--DDAVLRVFRLARVVRIFKLGKYAT	TVGYG	Abs.
112	Chr22465	---KAN---L	--LFRFLRMFRLLRLLRLLRLEQYVD	TVGYG	Pres.
113	Chr25304	GIQ---MFVG----AIVKS	ANSFGFVRIVRLVRVFRVFKFGRYSI	TVGYG	Abs.
114	Chr27069	---DVH---N	----LGFKIMRLLRLTKIIR------	KTPW-	Red.
115	Chr3027	---EVS---Y	LGLLRIVRIVRLLRLLKLARVSRLFK	SIGYG	Pres.
116	Chr40677	GLK---IFSD----ALKLS	AS---FMRAVRLVRVFRVFKSGRYQL	TVGYG	Abs.
117	Chr49074	VLK---VVAE----TMFRS	ATDDGSLRLLRIARLVRLLKLAKQMS	TVGYG	Abs.
118	Chr6780	---EVS---Y	ARSLSLLRVSRLLRLLRLTRLYRYLE	SIGYG	Pres.
119	Chr6793	SMI---VLMR----TLKRS	AGGLGVLRIVRLTRVLRVFKVSKSMQ	TVGYG	Abs.
120	Chr8707	GLL---VLLR----TLVKS	ADALAVLRIVRLTRVLRVVKKSKSLS	SEGYG	Abs.
121	Chr9301	ALI---VLWK----TIIRS	LGVLQILRLIRLTRISRIFKMSKKFQ	KVGYG	Abs.
122	Cryptog4458	TIE---VMYV----AVRRS	EDTFSILRTFRLLRVFRAFKHQNQML	TVGYG	Abs.
123	Danio4xD1	-EPDTN---K	--YMRF---LEASRCLRVFRLFTIIS	TIGYG	Pres.
124	Danio4xD2	-DLDLY---S	WTVSCL---VRLNRLLLIRRIYLYFR	SVGYG	Pres.
125	Danio4xD3	-DIAAA---T	LHFYAY---LRTPHILQLYRIPLLIS	TVGFG	Pres.
126	Danio4xD4	-SITSN---L	PIMIIY---ARIAHLPRIISLLVFMW	TVGFG	Pres.
127	Dros122	GLQ---ILGR----TLKAS	AMSLAILRVIRLVRVFRIFKLSRHSK	TVGYG	Abs.
128	Dros4470	---E-----Y	GSL---FSALKVVRLLRLGRVVRKLD	SVGFG	NI
129	Dros7256	NFLNMA---S	LRILRLAKLLSLVRLLRLSRLVRYVS	CIGYG	Pres.
130	Dros8960	-------GYP	-------CIVRLNRLLRINRLWEWFD	TI--G	Pres.
131	Emilih1782	ELQ---ALAA----CVRRC	PQFLTMVRVLRLVRILRLLRFAKESS	TVGYG	Abs.
132	Emilih183686	STV---LFKA----SL--S	PVQVAMLEVFRVVRILKIARNPDSTV	TVGYG	Abs.
133	Emilih21357	ELQ---LFAK----AISKS	ASGAGVLRVMRVVRLMRL----RHST	TVGYG	Abs.
134	Emilih5070	GCR---ILGQ----TLTES	PSALEMLQYYQFLSSLRLLKLTRHML	TVGYG	Abs.
135	Goniopac0043	RLR---PASF----SWWYS	FRLYLVIRVIRDRTDIYERRVYIIER	TTGYG	Abs.
136	Goniopac1159	RVR----------------	---LLVFRLVRVLRDASITRLIQRVR	TVGYG	Abs.
137	Goniopac2926	------LFF----------	--QVKMLRMVRMVRMVRIIKI----Q	TVGYG	Abs.
138	Goniopac360812	---KLN---P	IFVLKLLRLIRLLKM----------K	TVGYG	Pres.

139	Goniopac4299	HFQ---MIVK----TLSIS	FSQLRVLRSVRLFRVFRVFKLGKHSG	TVGYG	Abs.
140	Goniopac4884	---ALQNATL	--ARRYFRRFFLFRLLRLLRLAKVVK	TVGYG	Pres.
141	Hond1530	-TFDIN---P	LKALRF---MRVAKLIKVSKLRQLVS	SVGFG	Pres.
142	Hond3016	WMH---VFVA----TLQQS	----ISGGSFRAVRLVRIFRVLKVGR	TVGYG	Abs.
143	Hond4246	---------A	ATAINM---VKCIRLIRITKIFKFLE	SVGYG	Pres.
144	Hond5239	WMR---LYVN----AFTNA	-----SPQALRVARLMRVARVFKLSR	TVGYG	Abs.
145	Hond7007	EDRLKIRIHD	LKLIRLVRMTKLFRLLRMGRAAKFFA	GIGF-	Pres.
146	Hond7128	---------T	--YQVF---FPINKLARITRVIKRLD	TVGYG	Pres.
147	Hond8904	WIR---IFVV----AMQNS	-----DAAVFRVVRLVRVFRVFKISR	TVGYG	Abs.
148	Hond9142	---DITLS-V	LKVGRI---LKLAKLARLLKLPHLLG	TVGYG	Pres.
149	Hond9401	---RLK---P	SKLIRL---VKLTRLLRLVGLYRYFK	TVGYG	Pres.
150	Hs1185	TY-DLA---S	LRIVRFTKILSLLRLLRLSRLIRYIH	CIGYG	Pres.
151	Hs6036	-------NYP	---------IRLNRLLRFSRMFEFFQ	TI--G	Pres.
152	HsEAGREF	---E-----Y	SSL---FSSLKVVRLLRLGRVARKLD	SVGFG	Pres.
153	HsHCNREF	TY-DLA---S	LRIVRFTKILSLLRLLRLSRLIRYIH	CIGYG	Pres.
154	HsKvREF	GLQ---ILGQ----TLRAS	AMSLAILRVIRLVRVFRIFKLSRHSK	TVGYG	Abs.
155	KvAP	------KFLS----AIAAD	--GLGLFRLVRLLRFLRIL-------	TVGYG	Abs.
156	KvLm	PIY-----------SFFRS	---YGGFRAARIVSFVHLL-------	TVGYG	Abs.
157	LbiK	---VIN---P	TDSLKILRLLRSVRVFELFKSLRMLA	TIGYG	Pres.
158	Lingula4xD1	---DLSSN-A	WHLVGQ---LRMNRLLRFYKIPLFFD	STGYG	Pres.
159	Lingula4xD2	---GLEWS-L	WLIFRI---LRLNRLIKFWKVPHYFH	STGYG	Pres.
160	Lingula4xD3	---DIRRE-V	LNYLVF---LRLNRMINIYHLSLAFS	SVGYG	Pres.
161	Lingula4xD4	---ELNVS-M	VSMWSY---LRLLHLLRLVKVHQFFA	STGYG	Pres.
162	Lot0433	E-------------FFFGS	ADVLRAVQVFRVFRVLRFARLSEEF-	TVGYG	Abs.
163	Lot80703	DIH---TVPL----VIKQS	--SSESLRVLRLTRILRVVKIGRFNR	TLGYG	Abs.
164	Lot85122	GLL---LFYK----VIAKS	--GFQVLRVLRLTRAFRVFKLGKYSE	TVGYG	Abs.
165	Lot86777	---WLD---Y	LR---------LFRLLRLRRFFPFIT	TVGYG	Pres.
166	Lot9618	EIE---MALG----LPPSQ	--PLTVLRLLRVLRLQRYLKDFETFK	TVGFG	Abs.
167	Lottia3678	---E-----Y	STL---FSALKVVRLLRLGRVVRKLD	GVGFG	Pres.
168	Lottia4816	-FLAIA---G	LRMLRLVKLLSLLRLLRLSRLVRYVQ	CIGYG	Pres.
169	Lottia5598	-------SFP	---------LRLNRLLRIWRINEFYN	AI--G	Pres.
170	Lottia5762	GLQ---VLIM----TFRAS	STSLAFLRVIRLVRV---FKLTKHSA	TVGYG	Abs.
171	LpcK	---NVN---P	--PGYVIGIARLIRLLRGFQCLAIFR	TVGYG	Pres.
172	Mneml0466	--------SS	DSV---LYFLKAIAMLRIFRVTR-SS	TVGFG	Pres.
173	Mneml3742	-------SVP	LCMLDNLKLIRLSKFFKFHRLVAWND	TI--G	Pres.
174	Mneml9346	GLI---TLGL----ALRAS	LDSFVVLRVVRLARVFRILKLSRHSR	TVGYG	Abs.
175	Monbr6676	GLQ---DMIV----CISKS	ASSVAVVRILRLTRISRLLKFSRHSE	TVGYG	Abs.
176	Monbr9885	---NTRY--A	---------LRWNKFLRLPRVLERIQ	DVG-G	Pres.
177	Mortierel0503	TIE---VMIV----AIKRS	RDTFTILRLFRLLRVFRTFKYSSTIM	TTGYG	Abs.
178	Mortierel9832	TIE---VMIV----AIKRS	RDTFTILRLFRLLRVFRTFKYSSTIM	TTGYG	Abs.
179	MVP	N------------------	--GLRVINLLRILVLLRIIKL----R	TVGYG	Abs.
180	Orbicf0666	---NVT---R	LRILRMAKLLSLLRLLRISRLVRYVH	CIGYG	Pres.
181	Orbicf5061	GLQ---VLGY----TLRSS	VTSLAVLRVIRLVRVFRIFKLSRHSK	TVGYG	Abs.
182	Orbicf8378	-------SYP	-------PAIRMNRLIKYHRFWHFLD	TI--G	Pres.
183	Orbicf8450	---E-----Y	TTL---LGFLKVFRLLRLGRVARKID	TIGFG	Pres.
184	Ostr0014	RLS---IIAT----SLAEC	VSGFQILRMVRLVRVLRLVKLGSKFR	TVGYG	Abs.
185	Ostr0029	---RSN---L	NR----VRVLRLLRMYRVRRM----S	TVGYG	Pres.

186	Ostr0041	---SLD---Y	LRT---LRLLRLVKLVRAFRVSRLYK	TIGYG	Pres.
187	Ostr2923	KIQ---VVIT----AVRDS	ASGARLLRIFRLFRVIKVFRLGSRAK	TVGYG	Abs.
188	Ostr3585	---RV----Y	SKVPEVFRLFKMLRLLRVLRTMRILE	TVGYG	Pres.
189	Ostr4104	KIQ---VVIT----AVRDS	ASGARLLRIFRLFRVIKVFRLGSRAK	TVGYG	Abs.
190	Ostr6137	RLS---IIAT----SLAEC	VSGFQILRMVRLVRVLRLVKLGSKFR	TVGYG	Abs.
191	Ostr6492	KIQ---VVIT----AVRDS	ASGARLLRIFRLFRVIKVFRLGSRAK	TVGYG	Abs.
192	Ostr7909	---RAT---P	------ERFTRILRLLRVGKFGALFM	TVGFG	Pres.
193	Part0078	GVD---ILYK----SVADS	--GKTAFRILKLFKVIRILKVKRYLK	TVGYG	Abs.
194	Part1576	---KFN---Y	LKLPRMYRLFKIIRFIDMMKY--GIS	TVGYG	Pres.
195	Part1680	---DFSNSMI	LRILKFFRFIKVVRLLRVLKLKKIFS	TVGYG	Pres.
196	Part2060	---QFN---Y	LKLPRMYRLFKIIRFIDMMKY--GIS	TVGYG	Pres.
197	Part2164	---NLN---S	TRLPRMYKLISISRMWSTVQNNNCFS	TVGFG	Pres.
198	Part3746	---NITVTIQ	AKLPKAYKMIKLVKMSRMLK-KKFGE	TTGYG	Pres.
199	Part3991	---DISGSVV	LRILKFFRLIKIIRLLRVLKLKQLFN	TVGYG	Pres.
200	Part4007	---SDNSAIQ	LRVLKFFRFIKVIRLLRLAKLKVIVD	TVGYG	Pres.
201	Part5168	---FQDKTII	IKIMRIVRFIKVIRLMRALKLKKIIN	TIGYG	Pres.
202	Part5598	---SIEQTLQ	LKFVRLFKFIKIVRLLRVLKLGRILI	TLGYG	Pres.
203	Part5644	EIS---MLAD----TVKNS	--SLRVLRLIRFIRIFRLFKLSRFMK	TVGYG	Abs.
204	Part6349	---QTYTTIN	LRLLKFFRFIKIIRLLRLAKLKVIFD	TVGYG	Pres.
205	Part7227	DML---MIVD----TVKHS	--SLRVIRIIRFMRVFRLFKLSRFMK	TVGYG	Abs.
206	Part7798	---SLDKSIT	IKIMRILRFVKVIRLMRALKLKKIIN	TIGYG	Pres.
207	Part7972	DML---MIAD----TVKHS	--SLRIIRIVRFMRVFRLFKLSRFMK	TVGYG	Abs.
208	Part8397	GVK---ILIR----GLSES	----QVIKVLRMLRLIRIFKFSTFSL	TVGYG	Abs.
209	Part8423	---QLNIMLS	LRLVRFMRFIKIIRLLRLAKLKVFFD	TVGYG	Pres.
210	Part8681	DML---MIVD----TVRNS	--NLRVIRIIRFMRVFRLFKLSRFIK	TVGYG	Abs.
211	Part8783	---TLAQTFQ	FKIVRLFKFIKIIRLLRVLKISKILQ	TVGYG	Pres.
212	Part8867	---NIS---Q	AKLPKAYKMVKLIKMSRMLK-KKYGE	TTGYG	Pres.
213	Part9500	GVK---ILIR----GLSES	----WVIKVLRMLRLIRIFKFSTFSL	TVGYG	Abs.
214	Part9850	GVD---ILYK----SVADC	--GKTAFRILKLFKVIRILKIKRYLK	TVGYG	Abs.
215	Perkm7058	GWR---LLVI----SLSLS	-------------RMLKL---TRYST	TVGYG	Abs.
216	Perkm8060	GWR---LLVI----SLSLS	-------------RMLKL---TRYST	TVGYG	Abs.
217	Phyt5745	AIQ---VFTQ----AISIS	AGSFGAIRIVRLTRVARVLKMSRYSS	TVGYG	Abs.
218	Pm100515	IFT---LFTR----VISQS	SSALMVLRILRLTRIFRVFKLGKYNE	TVGYG	Abs.
219	Pm11858	---NPS--LS	LRS---LRVARIARCVRLLRLGLVGS	TVGYG	Red.
220	Pm124306	MLT-SAVFVE----VFRSG	QRTAHIIQVVRFCRVLRIAKAARHSQ	TVGYG	Abs.
221	Pm13328	---PMS---F	LKVIRL---FRLVRLLRLARVQRIFN	SIGYG	Pres.
222	Pm14163	LLS---IAV-----FIRVS	MRTLRMIRIVRLFRVMRIAKVARHSK	TVGYG	Abs.
223	Pm255449	------LLWH----TLKVA	--VVKSIDIILLVPVMRMIKA----R	TVGYG	Abs.
224	Pm256265	---SMK-EYQ	--YSTLLKLLRTPRLAPLVSM----K	TVGYG	Pres.
225	Pm258592	LLS---IAVL--GKVWRSS	GAAATILRIIRLIRILRVAKIARHST	TVGYG	Abs.
226	Pm260406	---NPG--LG	LRA---LRIARVVRCARLFRLGLLGS	TVGYG	NI
227	Pm30427	---GMS---Y	VKVVRLMRFLRLVRLLRLSKFQVIIA	SIGYG	Pres.
228	Pm31544	---NPG--LG	LRSMRTLRISQVARCFRLCRTYLLGN	TVGYG	Pres.
229	Pm35067	---GIS---N	VKIARL---LRLVRLLRLAKIQRISS	SIGYG	Red.
230	Pm35426	---SIS---Y	MKVIRL---MRLTRLLRLAKVQRLVN	SIGYG	Pres.
231	Pm44058	--------FI	TRVTKVMRFMRLIRLVKLYRL----Q	TIGYG	Pres.
232	Pm8705	---GIS---F	MKVLRL---LRLARLLRLAKVQKLVV	SIGYG	Pres.

233	Rhiz1414	*GFN----------TVFQRS*	*--SLKFIRVLLLLRVLRLFNVAKYTV*	*TTGYG*	Abs.
234	Rhiz1575	*GFL---IILLSS-------*	*---ISPFATLRFTRILRLTRISRKYD*	*TTGFG*	Abs.
235	Rhiz7733	*TIE---VMIV----AVKRS*	*HDTFTILRLFRLLRVFKAFKYSSTIM*	*TTGYG*	Abs.
236	Rhodm103319	*---DLR----*	*FHWSSTFRFLRLLRFYDIFRM----R*	*TVGYG*	Pres.
237	Rhodm14260	*---RVN----*	*LTWLSLLRFIRLVRVWEVLQA----R*	*TVGYG*	Pres.
238	Rhodm50364	*-----LLFGK*	*--GFQFVRLLRILRLQRLIQEDD---*	*TVGFG*	Pres.
239	Rhodm9830	*---KIS---V*	*LDFLLLFRVIRFRKKLRVIRF-----*	*TIGYG*	Pres.
240	Rhodomsp3660	*--------RP*	*LRLVRVLKPLRIFKMLRVIKVLHVFD*	*TVGFG*	Pres.
241	Rhodomsp4159	*GMW---LLSA----TMVRS*	*GGTTSFLRVIRLARVFRLFKLSKYSQ*	*TVGYG*	Abs.
242	Rhodomsp4271	*---RIS---P*	*LRFIRVVKPLRIVKLLRVFKFFDVLD*	*TVGFG*	Pres.
243	Rhodomsp4926	*-------RPP*	*LRLTRVLKPLRLSKMFRVLKISQYAE*	*TVGFG*	Pres.
244	Rhodomsp5962	*---RI----P*	*LRVIRALKPLKIARILRSIKFLKLVS*	*TVGFG*	Pres.
245	Rhodomsp6827	*---RLS---P*	*LRFVRVLKPIRLVKLLRIFKVFDILD*	*TVGFG*	Pres.
246	Saitoella2107	*GMK---VTFR----VFRRS*	*TAIFQVLRIFLLFRIVKVGQFTEWSI*	*TLGYG*	Abs.
247	Salp4xD1	*---RIGLS-Y*	*AHLARF---YRLNRLLRAAFLWQYAR*	*STGYG*	Pres.
248	Salp4xD2	*---ESFQR-N*	*MPVVAA---VRLNRLLRLHTAWSLLS*	*STGYG*	Pres.
249	Salp4xD3	*---NIAID-V*	*STVFCL---LRLNRCFRLARVMLFFG*	*SVGYG*	Pres.
250	Salp4xD4	*---ELDAS-V*	*FTAVSL---LRLTRVCRLFRAMHFFS*	*TTGYG*	Pres.
251	Salp1538	*---TFNLS-A*	*LKVLRMTRLLTLLRLLRVTRMFRLFS*	*CIGYG*	Pres.
252	SthK	*----IN---P*	*--PLSLLSLVRLLKLISV-------Q*	*TIGYG*	Pres.
253	Str0120	*---NSE-ALI*	*LR---IIKFYRLLRLIRLAKMKKVLV*	*TVGYG*	Pres.
254	Str0956	*---RLS---T*	*VRVSRIYKMLRMLRILKTLRLGVYIE*	*KIGYG*	Pres.
255	Str1302	*---ASN-TLA*	*LRLMRIFRFLKVLRLLRLAKLKKILM*	*TVGYG*	Pres.
256	Str1316	*---TIR---H*	*ARLPRLYKLFKISKVLKCLKQNRIMM*	*SVGYG*	Pres.
257	Str1416	*---DSE-AII*	*LR---VLKFYRLLRLIRLAKMKKIFI*	*TVGYG*	Pres.
258	Str1659	*---IIN---E*	*IRLLRLYRLIKIYHLIKQFKNRYGPH*	*TVGYG*	Pres.
259	Str2484	*---ASN-ALA*	*LRLVKIFRFMRILKLMRLTKLKRILM*	*TVGYG*	Pres.
260	Str2486	*---SSH-FLA*	*LR---ILRFYRILRLLRLAKLKKILI*	*TVGYG*	Pres.
261	Str2853	*---NIS---A*	*AKLPRVARIIRILRLIKVIKTKFWYN*	*TIGYG*	Pres.
262	Str2857	*---------K*	*IRFARIYRISKLLKLLKRFRVSDSLK*	*TVGYG*	Pres.
263	Str3241	*---NIN---H*	*FRLRSIPKLFRFSKAIKFIKTKTLLE*	*SVGYG*	Pres.
264	Str3433	*---NIN---H*	*FRLRSIPKLFRFSKAIKFIKTKTLLE*	*SVGYG*	Pres.
265	Str3516	*---QLN---A*	*SKLPRLYRLLRVFRIMKVLKHTQVLN*	*TVGYG*	Pres.
266	Str3541	*---VIN---E*	*IRLLRLYRLFKIYHLIKQFKNGNETQ*	*TVGYG*	Pres.
267	Str4014	*---KMT---E*	*ARIPRLYRIFRFLKLLKFMRIRSFFN*	*TIGFG*	Pres.
268	Str4420	*---REH---N*	*LRLPRLYKLFKVFRMNRIIRIKRFVK*	*TVGYG*	Pres.
269	Str4679	*---KVS---T*	*IRVSRIYKILRMVRILKTLRLRTYME*	*KIGYG*	Pres.
270	Str5032	*---RLA---T*	*SRMPRIYKVLRVVRLFKMVRLRSCIA*	*TVGFG*	Pres.
271	Str5197	*A-NETL---I*	*FSFFKFIRVLKLFKLVRLVKLRMLIF*	*SIGYG*	Pres.
272	Str5952	*---------K*	*VRFARIYRISKLFKLLKRFRVSNSLK*	*TVGYG*	Pres.
273	Str5999	*---SIK---H*	*LRLPRLYRLFRVVRLLKMFKHKNILE*	*TVGYG*	Pres.
274	Str6016	*---ASN-ALA*	*LRLVKIFRFMRILKLMRLTKLKRILM*	*TVGYG*	Pres.
275	Str6145	*---KVS---V*	*SRLPKLYRMIKIARLLRVMKLNNLLK*	*TVGYG*	Pres.
276	Str6239	*A-NQTL---I*	*FSFFKLVRVAKLLKLIRLAKLKILIF*	*SIGYG*	Pres.
277	Str6256	*---ASN-TLA*	*LRLVRMTRFLRVLRLLRLAKLKKILM*	*TVGYG*	Pres.
278	Str6710	*S-NKKI---L*	*LKYIRILRVLKLIKLIRLTKLKLMII*	*SIGYG*	Pres.
279	Str7755	*---ASN-TLA*	*LRLMRIFRFLKVLRLLRLAKLKKILM*	*TVGYG*	Pres.

280	Str7834	A-NQTL---I	FSFFKFIRVLKLFKLVRLVKLRMLIF	SIGYG	Pres.
281	TerK	---NIE---P	--SLFIIGLCRCPRLLRLPQFYRIFN	TVGYG	Pres.
282	Tfet5153	FL------------AFKEM	PLFWGVTYFSRIYRNRDEVQKAIETM	TVGYG	Abs.
283	Thalassm834790	NFR---MALG----MRRAD	--ALLNLRLLRILKFQRILTDSNTYM	TVGFG	Abs.
284	Thecat1176	---SAN---G	PKILRLLRMHRLLRLIQYVRSTRKFR	TVGYG	Pres.
285	Thecat1364	GFG---LVTQ----ALMAS	LGSFAVIRVVRLTRVFRLFKLSKYSK	TVGYG	Abs.
286	Thecat4561	---RVN---S	LRLLRILRLIRLVRMVRLFRLSRIYR	TTGYG	Pres.
287	Thecat5195	---DVN---S	LRVVRMIRLARLVRLLRIFRLSRLYR	SLGLG	Pres.
288	Thecat6466	-------SRP	------HAFMRLNRLLRLYHVPNIFR	GMGPD	Pres.
289	Thecat8941	--------KP	PDLAIVLRLLRLLRLTRVG---PMFS	GLGVD	Pres.
290	Thecat9688	-MM---MIGN----ALRRA	ASATNVLRAVRLIAIRRHAKS----S	TIGYG	Abs.
291	Thecat9915	RFL---SIVR----TLVTS	VAGLAVIRVVRLARVFRLFRLSKYNH	TVGYG	Abs.
292	Tvag6965	---NLVYY-S	-SAYIV---LSLLKVFRLNRCMIALH	AIGYG	Pres.
293	TrichKv	GLQ---TLGK----TFKSS	------VEALRVLRLARVLRIFKLSR	TVGYG	Abs.
294	Vitr5198	-------LLF	LQLLRIIKIFKLFRLVRLLKLLRIFT	TIGYG	Pres.
295	Vitr8094	GLR---LMGE----GLKRS	LGGFRVLRVVRLIRVFRIFKLSRYSV	TVGYG	Abs.
296	Vitr9089	--------SS	SRAFRMLKLIRLTKLMRIARMARSLQ	CIGYG	Pres.
297	Volvox0072	KLL---VVLH----SLRKS	FNGTTVFRVVRLLRVFRVLKLGGRYS	TVGYG	Abs.
298	Volvox2111	H-VRMS---Q	ASKIVWLKLMALSRAYRVFHV-----	SMGDG	Pres.
299	Volvox2132	NLQ---LV-----DALSAS	ASQTRIFRLIRLIRILRLLRASSRFR	TTGYG	Abs.
300	Volvox5751	---YAA---R	PRVRHLVVMLRLLRFFRIFKLSSVLG	TVGYG	Pres.
301	Volvox8978	--------------TLRDS	--QTRVVRLLRVFRVLRIMKV-----	TTGYG	Abs.
302	Volvox9510	---GAL---S	QRAVNALSLIRLIRLLRLVSVSKIYI	TTGFG	Pres.

Table S2. Functional determinants of K_v-like channels (yellow) and CNBD-channels (blue) considered in the analysis: S4–S5 linkers, voltage sensors S4, selectivity filters (SF), and cyclic nucleotide-binding domains (CNBD).

Arginine (R) and lysine (K) of S4s are highlighted in lilac. Abs. – absence of CNBD in the case of K_v-like channels; NI – not identified, probably due to incompleteness of an amino acid sequence in databases; Pres. – presence of CNBD; Red. – partial reduction of CNBD.

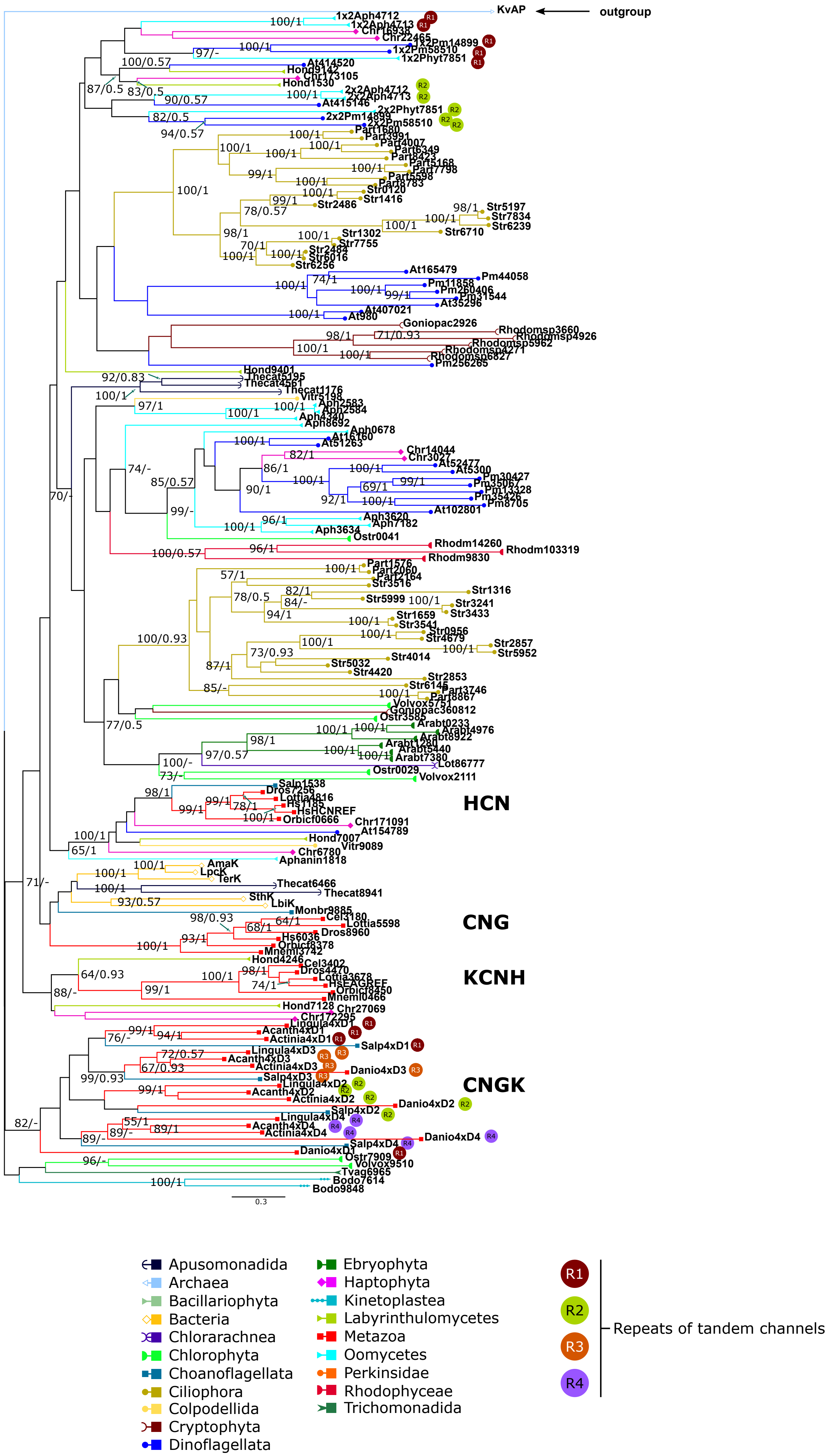
KvAP
outgroup
HCN
CNG
KCNH
CNGK
0.3
Apusomonadida
Archaea
Bacillariophyta
Bacteria
Chlorarachnea
Chlorophyta
Choanoflagellata
Ciliophora
Colpodellida
Cryptophyta
Dinoflagellata
Ebryophyta
Haptophyta
Kinetoplastea
Labyrinthulomycetes
Metazoa
Oomycetes
Perkinsidae
Rhodophyceae
Trichomonadida
R1
R2
R3
R4
Repeats of tandem channels

Figure S1. Maximal likelihood tree of CNBD-channels with KvAP (K_V-like channel of the archaeon *Aeropyrum pernix*) as an outgroup. LG + F + R7 model, 10000 ultrafast bootstrap replicates. The numbers on branches show bootstrap values and Bayesian posterior probabilities, respectively (not shown when supports < 70/0.90). For sequence abbreviations see Supplementary Table S1. The alignment is provided in Supplementary Dataset S3.

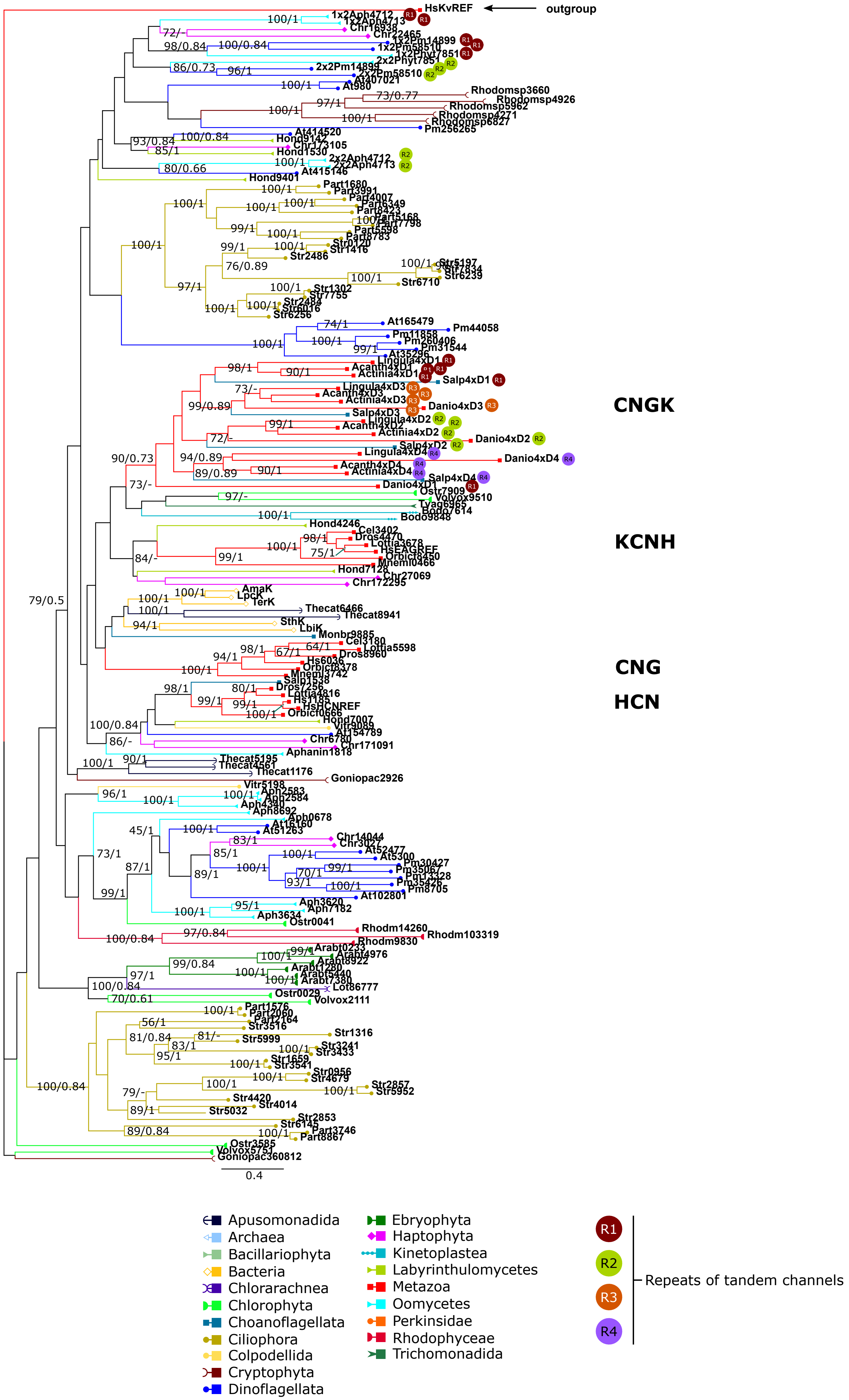

HsKvREF
outgroup
CNGK
KCNH
CNG
HCN
0.4
Apusomonadida
Archaea
Bacillariophyta
Bacteria
Chlorarachnea
Chlorophyta
Choanoflagellata
Ciliophora
Colpodellida
Cryptophyta
Dinoflagellata
Ebryophyta
Haptophyta
Kinetoplastea
Labyrinthulomycetes
Metazoa
Oomycetes
Perkinsidae
Rhodophyceae
Trichomonadida
R1
R2
R3
R4
Repeats of tandem channels

Figure S2. Maximal likelihood tree of CNBD-channels with HsKvREF (Kv1.7 channel of *Homo sapiens*) as an outgroup. LG + F + R7 model, 10000 ultrafast bootstrap replicates. The numbers on branches show bootstrap values and Bayesian posterior probabilities, respectively (not shown when supports < 70/0.90). For sequence abbreviations see Supplementary Table S1. The alignment is provided in Supplementary Dataset S4.

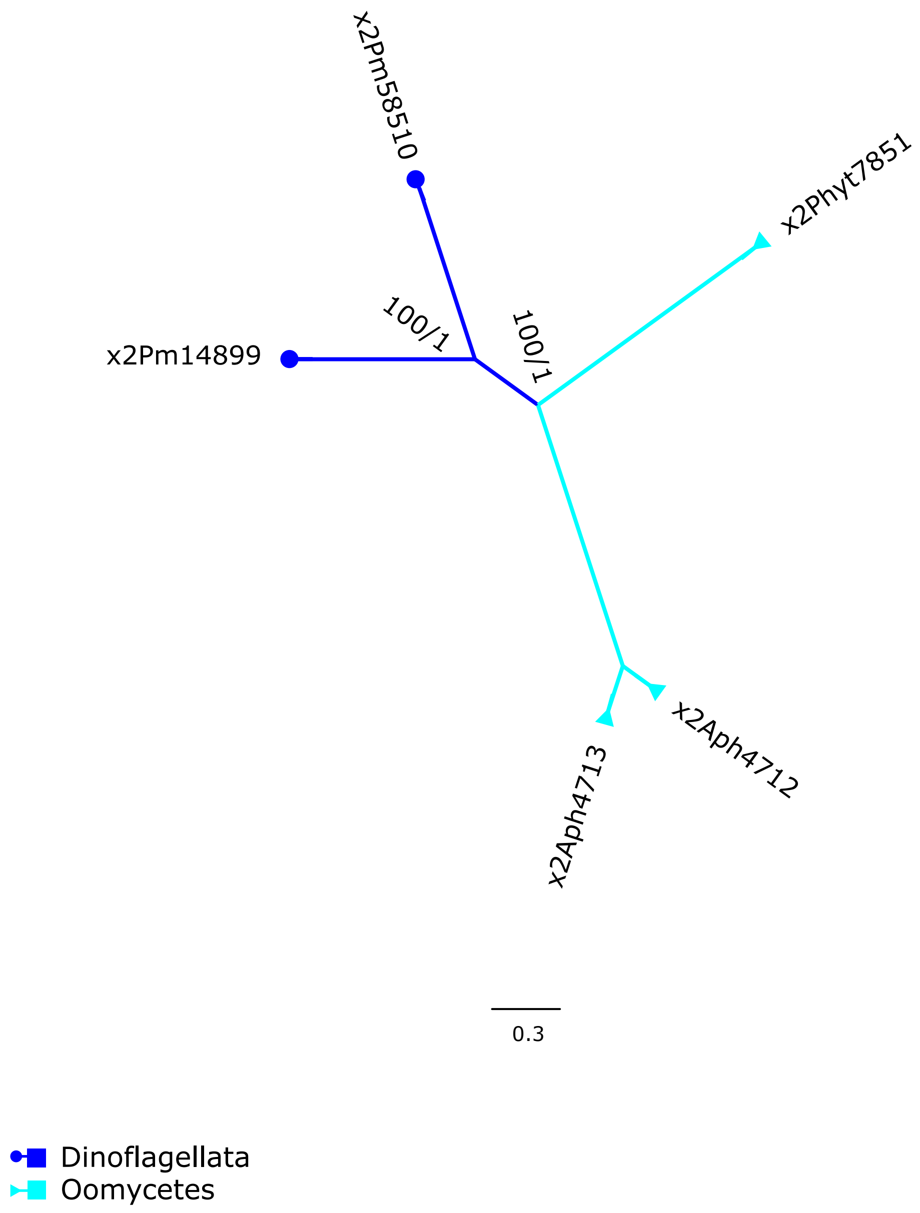

Figure S3. Unrooted phylogenetic tree of the two-repeat tandem CNBD-channels inferred using maximal likelihood analysis (LG + Γ_4 model, 10000 ultrafast bootstrap replecates). The numbers on branches show bootstrap values and Bayesian posterior probabilities, respectively (not shown where supports < 70/0.90). The alignment is provided in Supplementary Dataset S5. x2Aph4712 - *Aphanomyces invadans* (NCBI, XP_008874712.1), x2Aph4713 - *A. invadans* (NCBI, XP_008874713.1), x2Phyt7851 - *Phytophthora infestans* (NCBI, XP_002907851.1), x2Pm14899 - *Prorocentrum minimum* (MMETSP, Prorocentrim minimum CCMP2233, 14899_1), x2Pm58510 - *P. minimum* (MMETSP, Prorocentrim minimum CCMP2233, 58510_1).

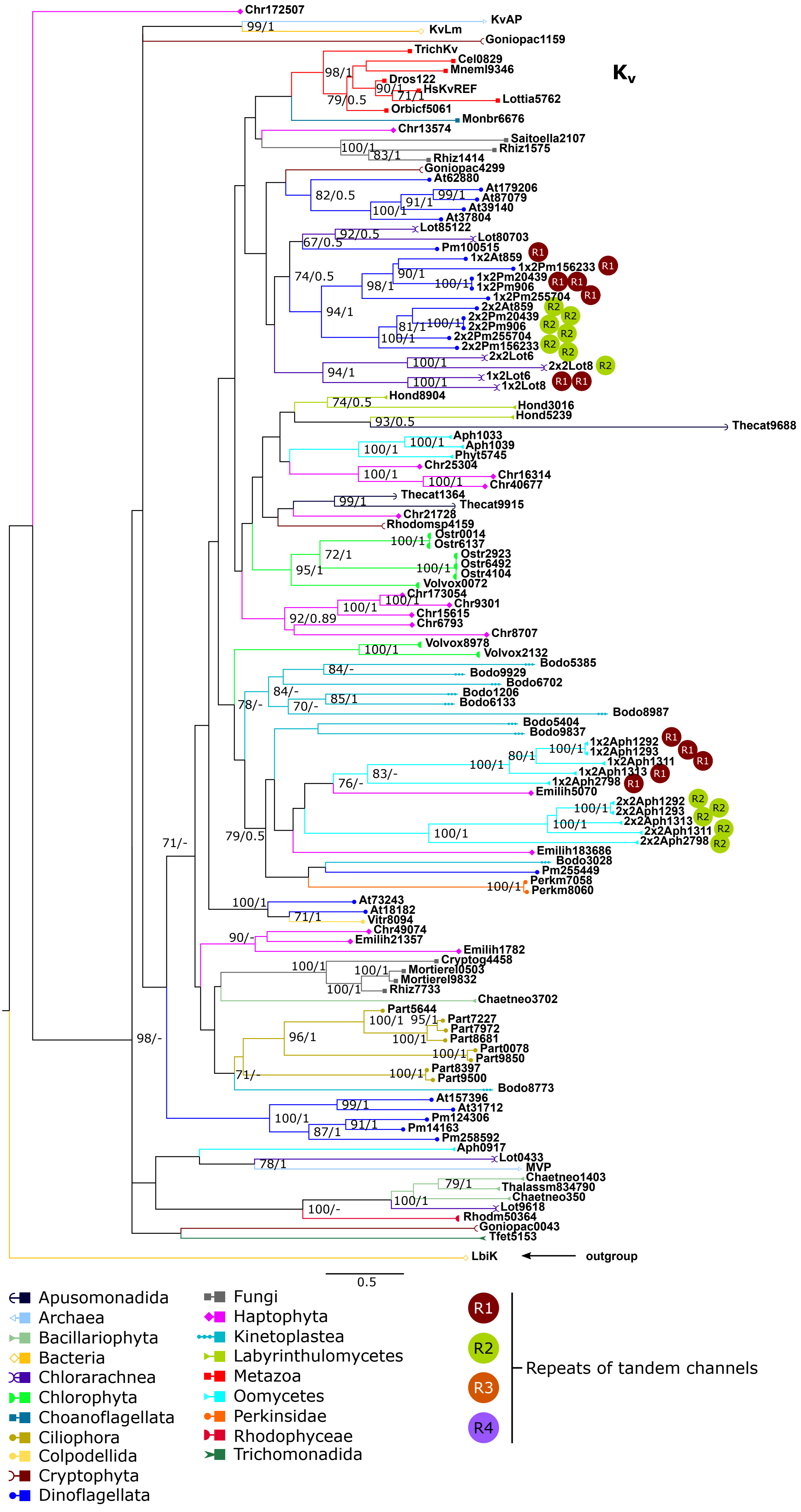
Kv
Chr172507
KvAP
KvLm
Goniopac1159
TrichKv
Cel0829
Mneml9346
Dros122
HsKvREF
Lottia5762
Orbicf5061
Monbr6676
Chr13574
Saitoella2107
Rhiz1575
Rhiz1414
Goniopac4299
At62880
At179206
At87079
At39140
At37804
Lot85122
Lot80703
Pm100515
1x2At859
1x2Pm156233
1x2Pm20439
1x2Pm906
1x2Pm255704
2x2At859
2x2Pm20439
2x2Pm906
2x2Pm255704
2x2Pm156233
2x2Lot6
2x2Lot8
1x2Lot6
1x2Lot8
Hond8904
Hond3016
Hond5239
Thecat9688
Aph1033
Aph1039
Phyt5745
Chr25304
Chr16314
Chr40677
Thecat1364
Thecat9915
Chr21728
Rhodomsp4159
Ostr0014
Ostr6137
Ostr2923
Ostr6492
Ostr4104
Volvox0072
Chr173054
Chr9301
Chr15615
Chr6793
Chr8707
Volvox8978
Volvox2132
Bodo5385
Bodo9929
Bodo6702
Bodo1206
Bodo6133
Bodo8987
Bodo5404
Bodo9837
1x2Aph1292
1x2Aph1293
1x2Aph1311
1x2Aph1313
1x2Aph2798
Emilih5070
2x2Aph1292
2x2Aph1293
2x2Aph1313
2x2Aph1311
2x2Aph2798
Emilih183686
Bodo3028
Pm255449
Perkm7058
Perkm8060
At73243
At18182
Vitr8094
Chr49074
Emilih21357
Emilih1782
Cryptog4458
Mortierel0503
Mortierel9832
Rhiz7733
Chaetneo3702
Part5644
Part7227
Part7972
Part8681
Part0078
Part9850
Part8397
Part9500
Bodo8773
At157396
At31712
Pm124306
Pm14163
Pm258592
Aph0917
Lot0433
MVP
Chaetneo1403
Thalassm834790
Chaetneo350
Lot9618
Rhodm50364
Goniopac0043
Tfet5153
LbiK
outgroup
0.5
Apusomonadida
Archaea
Bacillariophyta
Bacteria
Chlorarachnea
Chlorophyta
Choanoflagellata
Ciliophora
Colpodellida
Cryptophyta
Dinoflagellata
Fungi
Haptophyta
Kinetoplastea
Labyrinthulomycetes
Metazoa
Oomycetes
Perkinsidae
Rhodophyceae
Trichomonadida
R1
R2
R3
R4
Repeats of tandem channels

Figure S4. Maximal likelihood tree of K_V-like channels with LbiK (CNBD-channel of the bacterium *Leptospira biflexa*) as an outgroup. LG + F + R5 model, 10000 ultrafast bootstrap replicates. The numbers on branches show bootstrap values and Bayesian posterior probabilities, respectively (not shown when supports < 70/0.90). For sequence abbreviations see Supplementary Table S1. The alignment is provided in Supplementary Dataset S7.

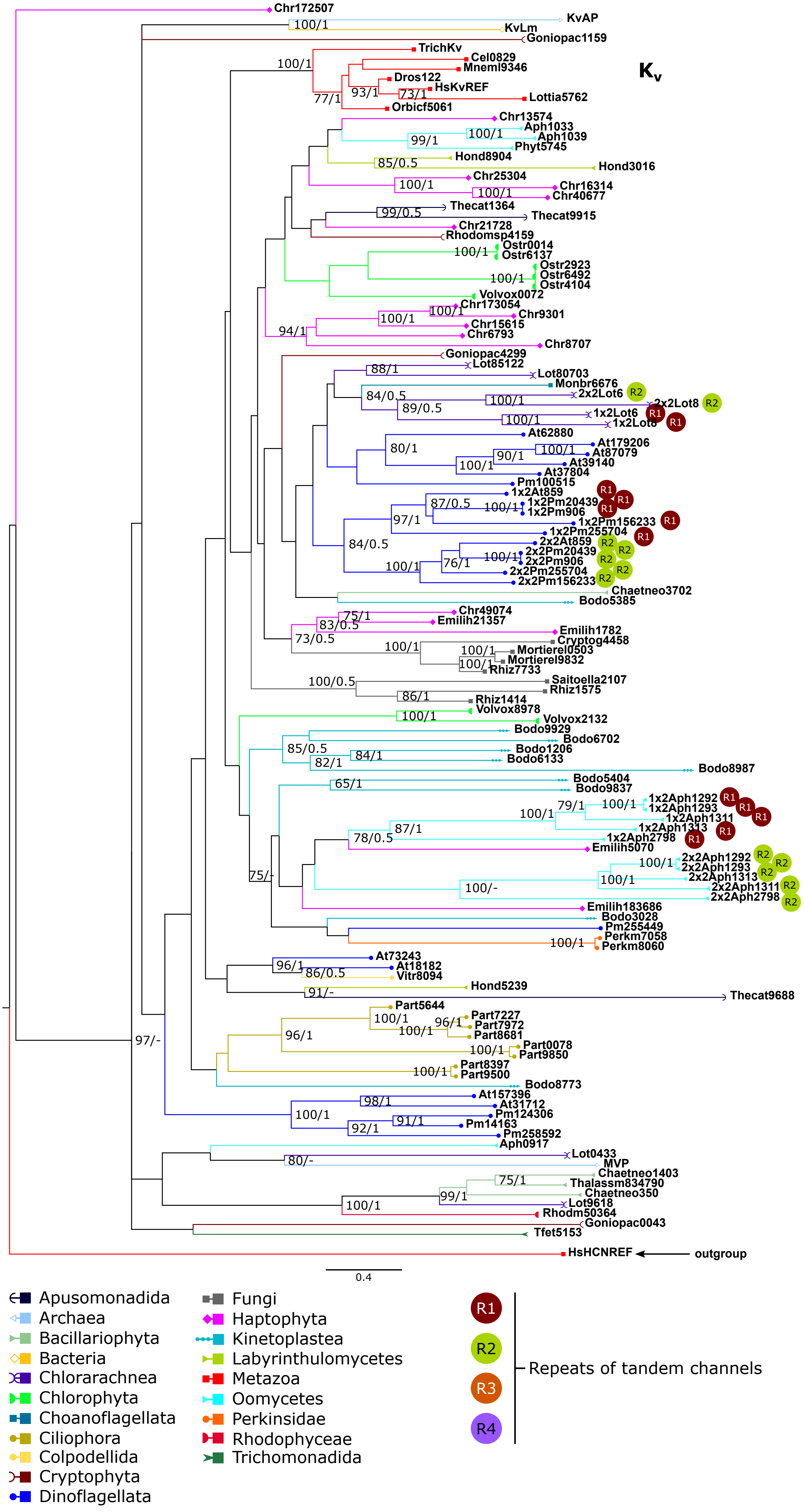

Kv
Chr172507
KvAP
KvLm
Goniopac1159
TrichKv
Cel0829
Mneml9346
Dros122
HsKvREF
Lottia5762
Orbicf5061
Chr13574
Aph1033
Aph1039
Phyt5745
Hond8904
Hond3016
Chr25304
Chr16314
Chr40677
Thecat1364
Thecat9915
Chr21728
Rhodomsp4159
Ostr0014
Ostr6137
Ostr2923
Ostr6492
Ostr4104
Volvox0072
Chr173054
Chr9301
Chr15615
Chr6793
Chr8707
Goniopac4299
Lot85122
Lot80703
Monbr6676
2x2Lot6
2x2Lot8
1x2Lot6
1x2Lot8
At62880
At179206
At87079
At39140
At37804
Pm100515
1x2At859
1x2Pm20439
1x2Pm906
1x2Pm156233
1x2Pm255704
2x2At859
2x2Pm20439
2x2Pm906
2x2Pm255704
2x2Pm156233
Chaetneo3702
Bodo5385
Chr49074
Emilih21357
Emilih1782
Cryptog4458
Mortierel0503
Mortierel9832
Rhiz7733
Saitoella2107
Rhiz1575
Rhiz1414
Volvox8978
Volvox2132
Bodo9929
Bodo6702
Bodo1206
Bodo6133
Bodo8987
Bodo5404
Bodo9837
1x2Aph1292
1x2Aph1293
1x2Aph1311
1x2Aph1313
1x2Aph2798
Emilih5070
2x2Aph1292
2x2Aph1293
2x2Aph1313
2x2Aph1311
2x2Aph2798
Emilih183686
Bodo3028
Pm255449
Perkm7058
Perkm8060
At73243
At18182
Vitr8094
Hond5239
Thecat9688
Part5644
Part7227
Part7972
Part8681
Part0078
Part9850
Part8397
Part9500
Bodo8773
At157396
At31712
Pm124306
Pm14163
Pm258592
Aph0917
Lot0433
MVP
Chaetneo1403
Thalassm834790
Chaetneo350
Lot9618
Rhodm50364
Goniopac0043
Tfet5153
HsHCNREF
outgroup
0.4
Apusomonadida
Archaea
Bacillariophyta
Bacteria
Chlorarachnea
Chlorophyta
Choanoflagellata
Ciliophora
Colpodellida
Cryptophyta
Dinoflagellata
Fungi
Haptophyta
Kinetoplastea
Labyrinthulomycetes
Metazoa
Oomycetes
Perkinsidae
Rhodophyceae
Trichomonadida
R1
R2
R3
R4
Repeats of tandem channels

Figure S5. Maximal likelihood tree of K_v-like channels with HsHCNREF (HCN1 channel of *Homo sapiens*) as an outgroup. LG + F + R5 model, 10000 ultrafast bootstrap replicates. The numbers on branches show bootstrap values and posterior probabilities, respectively (not shown when supports < 70/0.90). For sequence abbreviations see Supplementary Table S1. The alignment is provided in Supplementary Dataset S8.

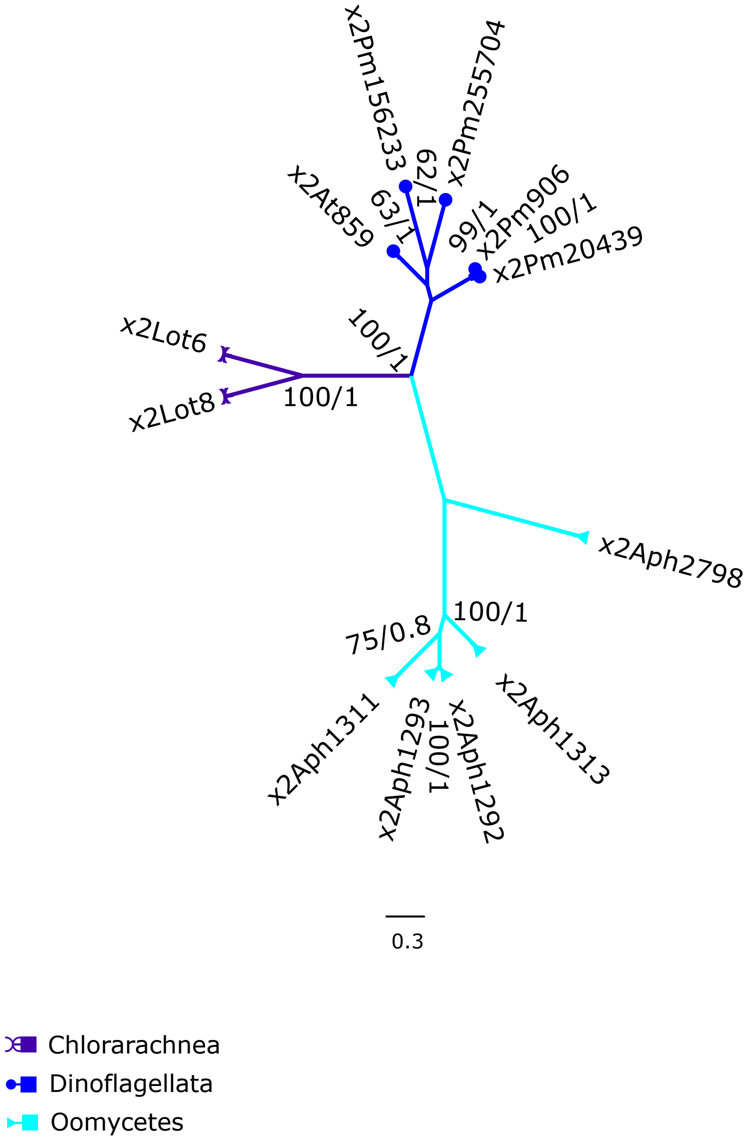

Figure S6. Unrooted phylogenetic tree of the two-repeat tandem K_V-like channels inferred using maximal likelihood analysis (LG + F + Γ_4 model, 10000 ultrafast bootstrap replecates). The umbers on branches show bootstrap values and Bayesian posterior probabilities, respectively (not shown where supports < 70/0.90). The alignment is provided in Supplementary Dataset S9. x2Aph1292 - *Aphanomyces invadans* (NCBI, XP_008861292.1), x2Aph1293 - *A. invadans* (NCBI, XP_008861293.1), x2Aph1311 - *A. invadans* (NCBI, XP_008861311.1), x2Aph1313 - *A. invadans* (NCBI, XP_008861313.1), x2Aph2798 - *A. invadans* (NCBI, XP_008862798.1), 2xAt859 - *Alexander tamarense* (MMTESP, Alexander tamarense CCMP1771, 859_1), x2Lot6 - *Lotharella globosa* (MMETSP, Lotharella globosa CCCM811, 116467_1), x2Lot8 - *L. globosa* (MMETSP, Lotharella globosa CCCM811, 81223_1), x2Pm156233 - *Prorocentrum minimum* (MMETSP, Prorocentrum minimum CCMP2233, 156233_1), x2Pm20439 - *P. minimum* (MMETSP, Prorocentrum minimum CCMP2233, 20439_1), x2Pm25574 - *P. minimum* (MMETSP, Prorocentrum minimum CCMP2233, 25574_1), x2Pm906 - *P. minimum* (MMETSP, Prorocentrum minimum CCMP2233, 906_1).
